# Decoding neuronal vulnerability: Multidimensional analysis of D1R‐ and D2R‐ medium‐sized spiny neurons in Huntington's disease

**DOI:** 10.1111/bpa.70135

**Published:** 2026-08-26

**Authors:** Guendalina Bergonzoni, Miguel Pellegrini, Aurora Savino, Martina Lazioli, Sarah Geurs, Lorenzo Graziani, Denise Ferrarini, Solaleh Khoramian Tusi, Esaria Oliver, Jarne Geurts, Sara Vinciguerra, Michele Tebaldi, Takshashila Tripathi, Isabella Pesce, Valeria Poli, Alessandro Romanel, Rosario Moratalla, Thierry Voet, Jasmin Morandell, Remo Sanges, Vanessa C. Wheeler, Erik Dassi, Marta Biagioli

**Affiliations:** ^1^ Laboratory of NeuroEpigenetics, Department of Cellular, Computational and Integrative Biology (CIBIO) University of Trento Trento Italy; ^2^ Molecular Biotechnology Center, Department of Molecular Biotechnology and Health Sciences University of Turin Turin Italy; ^3^ Department of Human Genetics KU Leuven Leuven Belgium; ^4^ Computational Genomics Laboratory International School for Advanced Studies (SISSA) Trieste Italy; ^5^ Center for Genomic Medicine Massachusetts General Hospital Boston Massachusetts USA; ^6^ Department of Neurology Massachusetts General Hospital and Harvard Medical School Boston Massachusetts USA; ^7^ Cell Analysis and Separation Core Facility, Department of Cellular, Computational and Integrative Biology (CIBIO) University of Trento Trento Italy; ^8^ Laboratory of Bioinformatics and Computational Genomics, Department of Cellular, Computational and Integrative Biology (CIBIO) University of Trento Trento Italy; ^9^ Cajal Institute, Spanish National Research Council (CSIC) Madrid Spain; ^10^ Laboratory of RNA Regulatory Networks, Department of Cellular, Computational and Integrative Biology (CIBIO) University of Trento Trento Italy

**Keywords:** D1R, D2R, differential vulnerability, transcriptomic analyses, Huntington's disease, medium‐sized spiny neurons (MSNs)

## Abstract

Understanding the molecular mechanisms driving selective neuronal vulnerability to different neurodegenerative disorders remains a crucial, unsolved question. Here, we explored the case of Huntington's disease (HD), where the striatum and, specifically, dopamine receptor 1 (D1R) and dopamine receptor 2 (D2R) medium‐sized spiny neurons (MSNs) exhibit differential susceptibility to the *HTT* CAG‐repeat expansion mutation, with D2R‐neurons being impacted earlier and more significantly. To unravel differences between D2R and D1R MSNs, we employed a multidimensional approach, integrating genomic, transcriptional, pattern distribution, and somatic instability analyses. Specifically, we used *Htt* CAG knock‐in mouse models harboring 18 (*Htt* ^*Q20*^: “control”) or ~190 (*Htt* ^*Q175*^: “HD”) consecutive CAG repeats, expressing tdTomato and EGFP under the control of *Drd1* and *Drd2* promoters, respectively. First, comprehensive genomic and transcriptomic analyses following fluorescence‐activated cell sorting (FACS) of dissociated striatal neurons revealed distinct gene expression profiles with a significant upregulation of oxidative phosphorylation and translation pathways in D1R‐positive neurons already at the pre‐symptomatic stage. These transcriptional changes were not accompanied by major copy number variations, as shown by parallel genomic analysis. Secondly, histological analyses revealed a greater proportion of D1R‐positive neurons compared to D2R‐positive neurons in HD mice, particularly in the ventral‐medial neostriatum, with D2R‐positive neurons presenting an increased nuclear accumulation of mutant huntingtin aggregates. In summary, our integrative study suggests that the distinct vulnerability of MSNs in HD might result from a combination of an early transcriptional compensatory response of D1R neurons together with specific susceptibility of D2R neurons.

## Graphical abstract

D1R and D2R MSNs show distinct responses to Huntington's pathology. G&T‐sequencing of pre‐symptomatic MSNs reveals global LINE‐1 downregulation. While D1R‐MSNs display early transcriptomic changes (OXPHOS/translation upregulation) and reduced mutant huntingtin aggregation, D2R‐MSNs exhibit higher nuclear mHTT accumulation and greater CAG somatic instability, supporting their preferential susceptibility to Huntington's pathology.

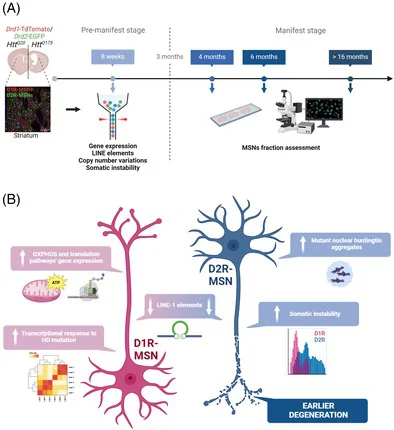

## 1 INTRODUCTION

Medium‐sized spiny neurons (MSNs) are the predominant inhibitory GABAergic neurons in the striatum, constituting approximately 95% of the neuronal population [1]. These neurons receive glutamatergic, excitatory inputs from the thalamus and the cortex, serving as the primary output of the striatum [2]. Notably, the activity of MSNs is modulated by dopamine, and depending on the expression of either dopamine receptor 1 (D1R) or 2 (D2R), MSNs elicit opposing effects on cAMP production [3] in the globus pallidus (GP), thereby regulating its activation and influencing voluntary movement control. Specifically, in the direct pathway, D1R‐MSNs inhibit the GP internal segment (GPi), disinhibiting the motor thalamus and promoting movement. In the indirect pathway, D2R‐MSNs inhibit GP external segment neurons, leading to subthalamic nucleus (STN) activation, which excites the GPi and suppresses thalamic activity. Dopamine from the substantia nigra pars reticulata modulates both pathways in the striatum [1, 4]. Due to their fundamental role in the regulation of movement, alterations in MSNs are implicated in neurodegenerative diseases such as Parkinson's disease [5] and Huntington's disease (HD) [6, 7, 8].

HD, specifically, represents a prototype monogenic, neurodegenerative disorder distinguished by motor, cognitive, and psychiatric symptoms, as initially described by George Huntington in 1872 [9]. This lethal autosomal dominant disease is caused by an abnormal expansion of the CAG tract in exon 1 of the HD gene (*HTT*), responsible for encoding the huntingtin protein [10]. Currently, no cure is available for HD.

The striatum is the primary brain region that degenerates in HD. However, as the disease progresses, additional areas such as the cerebral cortex, subcortical white matter, thalamus, and hypothalamic nuclei become affected [11]. Striatal atrophy primarily results from the degeneration of MSNs [6, 7, 8]. Intriguingly, D1R‐ and D2R‐expressing MSNs exhibit distinct vulnerabilities in HD, with D2R declining earlier than D1R neurons [12]. Consequently, MSNs targeting the GPe experience significant loss at the disease onset, disrupting the balance between direct and indirect pathways. The absence of inhibition by D2R‐MSNs on the GPe leads to excessive activation of pallidal neurons, contributing to the observed choreiform movements in HD. However, D1R‐MSNs decline follows the initial D2R MSNs degeneration, impacting the GPi within the direct pathway and resulting in akinetic movements and rigidity that replaces the initial chorea [1, 13].

Despite these insights, the precise molecular mechanisms underpinning the distinct vulnerabilities of D1R‐ and D2R‐MSNs in HD remain incompletely understood [14]. Molecular sensitizers or compensatory mechanisms, potentially acting since pre‐symptomatic and early stages of the disease, might contribute to the differential sensitivity to neurodegeneration, but their identities and roles remain unclear.

While earlier cellular and molecular studies in HD models, such as the ones employing *Htt* ^*Q175*^ knock‐in mice, revealed morphological and electrophysiological differences between D1R and D2R neurons, with altered dendritic remodeling and excitability in D1R neurons [15], as well as enhanced glutamatergic input and vulnerability in D2R neurons [16, 17]—these approaches offered limited insights into the molecular heterogeneity underlying the observed changes. The advent of single‐cell genomics has enabled a more refined, transcription‐level view, revealing cell‐type‐specific responses previously overlooked. For example, recent work uncovered downregulation of oxidative phosphorylation and upregulation of neurotrophin signaling in D2R neurons in HD models, pointing to distinct molecular trajectories of degeneration [18].

In *post mortem* HD brains, both D1R and D2R‐MSNs display high levels of somatic *HTT* CAG expansion [19, 20]—a key driver of pathology—modulated by mismatch repair (MMR) genes [21, 22, 23, 24, 25, 26, 27]. Once the repeat surpasses a critical threshold, rapid degeneration of MSNs is triggered [19]. Moreover, somatic genomic mosaicism driven by LINE‐1 retrotransposition may add another layer of genomic instability. While decreased LINE‐1 RNA expression has been observed in the HD bulk striatum [28], its specific role in MSNs remains unexplored, while promising a better understanding of cell‐type‐specific genomic, epigenomic and transcriptional dysregulation in HD.

Thus, though significant advancements have been made in unraveling the intricate responses of D1R and D2R expressing MSNs to HD, an integrative, comprehensive analysis addressing the underlying molecular mechanisms driving their divergent vulnerabilities remains unavailable. This study aims to fill this gap by employing a multidimensional approach combining single‐cell genomic and transcriptomic analysis, pattern distribution studies and characterization of somatic mosaicism.

## 2 MATERIALS AND METHODS

### 2.1 Mouse models

*Tg(Drd1a‐TdTomato)6Calak* BAC transgenic mice expressing tandem‐dimer Tomato (TdTomato) under the control of *Drd1a* gene promoter [29] and *Tg(Drd2‐Egfp)S118Gsat* transgenic mice expressing EGFP under the control of *Drd2* gene promoter [30] were gently provided by Dr. Rosario Moratalla (Institute Cajal, CSIC, Madrid). Hemizygous animals from each strain were crossed to obtain double transgenic mice (*Drd1‐*TdTomato and *Drd2*‐EGFP) at the expected ratio of 25% per litter. Mice were maintained on a C57BL/6J background.

The *Hdh* ^*Q20*^ (now *Htt* ^*Q20*^) knock‐in mouse, generated by Dr. Marcy E. MacDonald (Massachusetts General Hospital), was created by inserting a chimeric mouse: human exon 1 with 18 CAG repeats into the murine *Htt* gene, resulting in a model with no pathological phenotype used as control [31, 32]. In contrast, the zQ175 (*Htt* ^*Q175*^) line, developed by Drs. Scott Zeitlin (University of Virginia) and Marie‐Francoise Chesselet (University of California, Los Angeles), was derived from a spontaneous expansion in CAG_140_ knock‐in mice, carries approximately 175 CAG repeats, and shows a pronounced phenotype suitable for studying pathogenic events in HD [33, 34].

*Htt* ^*Q20*^ knock‐in mice Stock No. [35] [CHDI‐81003005] and zQ175 knock‐in mice [36] [CHDI‐81003003] were purchased from the Jackson Laboratory.

For our experiments, matings were set up with a male double hemizygous positive for *Drd1‐*TdTomato and *Drd2*‐EGFP and a female positive for *Htt* ^*Q175*^. *Htt* ^*Q175*^ positive males were prevented from mating in order to avoid *Htt* meiotic instability, which would lead to a CAG tract size expansion in the progeny [37, 38]. Instead, males or females double hemizygous positive for *Drd1‐*TdTomato and *Drd2*‐EGFP were crossed with males or females positive for *Htt* ^*Q20*^. Mouse models of interest are triple transgenic, having the three alleles in heterozygosity or hemizygosity. Since in preliminary analyses no evidence for sex‐specific differences could be observed (data not shown), both sexes were combined and considered together.

All mice were housed in controlled conditions of temperature, which was maintained constant at 20–22°C, and 12‐h light–dark cycles, in individually‐ventilated cages (IVC—Techniplast GreenLine) and pathogen free (SPF), according to the FELASA 2014 Guide Lines [39]. Food and water were provided ad libitum. Mice were checked daily. Animals were euthanized with CO_2_ infusion and cervical dislocation, according to the D. Lgs. 26, 2014. All experiments were in concordance with the guidelines of the University of Trento and Italian Ministry of Health (nr.781/2016‐PR, 39/2023‐PR).

### 2.2 Molecular genotyping

An ear biopsy of 1–2 mm was collected from each animal to be genotyped. DNA was extracted using lysis solution (25 mM NaOH, 0.2 mM EDTA) followed by incubation for 1 h at 92°C and finally adding neutralizing solution (40 mM Tris HCl pH 5.5).

After DNA extraction, DNA samples from each mouse were genotyped by PCR. At first, positivity for TdTomato and EGFP was analyzed. TdTomato and EGFP genotyping PCRs were performed using the 2X Phusion Green Master Mix (ThermoFisher, #F‐566). Double positive samples were then tested by knock‐in PCR, in order to assess the positivity for the transgenic CAG tract. Knock‐in PCRs were performed using Taq PCR Core Kit (QIAGEN, #201223). For each reaction, positive and negative controls were included. PCR protocols followed manufacturer's instructions, with 30–35 amplification cycles. Primers used are reported in Table 1. Amplified samples were then run on agarose gel.

**TABLE 1:** Primer sequences used for the molecular genotyping of mice.

Primers	FW sequence	RV sequence	Amplicon
TdTomato	CTTCTGAGGCGGAAAGAACC	TTTCTGATTGAGGAGAGCATTCG	750 bp
Internal control	CTAGGCCACAGAATTGAAAGATCT	GTAGGTGGAAATTCTAGCATCATCC	324 bp
EGFP	GAGGAAGCATGCCTTGAAAA	TGGTGCAGATGAACTTCAGG	300 bp
*Hdh CAG1* (Q20)	ATGAAGGCCTTCGAGTCCCTCAAGTCCTTC	GGCGGCTGAGGAAGCTGAGGA	140 bp
*Neo* (Q175)	CTTGGGTGGAGAGGCTATTC	AGGTGAGATGACAGGAGATC	280 bp

*Note*: The table reports all the sequences of forward (FW) and reverse (RV) primers used for the molecular genotyping of mouse models and the expected amplicon lengths.

### 2.3 Perfusion and brain dissection

Triple‐positive mice selected for MSNs pattern distribution analysis (4, 6, and >16 months of age) were perfused and the brains were removed. Mice were anesthetized by an intraperitoneal injection of Ketamine (150 mg/kg) and Rompun (15 mg/kg xylazine). Then, perfusion was first performed with 1X PBS (Gibco, #70013016) to wash out the blood, followed by freshly prepared 4% FA (Formaldehyde solution, methanol‐free, ThermoFisher, #28908). After perfusion, the head was removed and craniotomy was performed. The brain region containing the striatum was coronally sectioned using a coronal brain matrix (Ted Pella, #15050). The sample was then post‐fixated in 4% FA at room temperature (RT) for 3–4 h. To cryopreserve tissues, the brain sample was then transferred to a 30% sucrose solution (in 1X PBS) at 4°C overnight.

### 2.4 Embedding and cryostat cutting

After sucrose incubation, the brain sample was transferred to a plastic embedding mold (Bio‐Optica, #07‐MP1515), embedded in OCT resin (Surgipath, FSC 22 Clear, Leica, #3801480), and incubated in dry ice until complete freezing. 16 μm brain sections were then collected on glass slides (Superfrost Plus, Thermofisher, #J1800AMNZ) using cryostat (Leica CM 1850 UV), according to bregma position from 0.145 to 0.845 mm.

### 2.5 Immunofluorescence

Striatal sections were further processed for immunofluorescence or nuclear staining. At first, sections were incubated in blocking buffer (10% foetal bovine serum (FBS), 5% bovine serum albumin (BSA) in 1X PBS) for 1 h at RT in a humidity chamber. EM48 (Merck Millipore, MAB5374), NeuN (GTX132974), or Darpp32 (GTX133336) primary antibodies were then diluted 1:100 in diluent solution (5% BSA, 0.1% Triton, in 1X PBS) and incubated overnight at 4°C in the humidity chamber. The following day, sections were washed 3 times in 1X PBS. Alexa Fluor 647 (#A21235 or #A21245) secondary antibody was diluted 1:500 in diluent solution and incubated for 1 h at RT in the humidity chamber.

For nuclear staining, sections were washed 3 times in 1X PBS and then incubated with TO‐PRO™‐3 Iodide (642/661) (ThermoFisher, #T3605) diluted 1:1000 in 1X PBS for 20 min at RT. Moreover, EM48 localization in the nuclei of the cells was verified checking its colocalization with Hoechst 33342 (ThermoFisher, #62249), which was diluted 1:5000 in 1X PBS and incubated for 5 min at RT (Figure 2E). EM48 absence of signal was verified in *Htt* ^*Q20*^ animals.

After immunostaining or nuclear staining, sections were washed 3 times in 1X PBS. Glass slides were mounted with FluorSave™ Reagent (Merck Millipore, #345789) and coverslip (Prestige™, #372114).

### 2.6 Imaging

Mouse striatal sections were visualized using the Leica TSC SP8 microscope. The following parameters were set up and applied for samples imaging: Light source: Lumencor SOLA‐SMII; Laser lines: Diode (405 nm), Argon (458, 488, 514 nm), Solid State (561 nm) and He/Ne (633 nm); Camera: Andor iXon Ultra 888; Scan heads: PMT and HyD; Objectives: HC PL FLUOTAR 10×/0.30 and HC PL APO 20×/0.75 CS2; Filters: Dapi (beam splitter: 425), FITC (beam splitter: 495), RHOD (beam splitter: 565) and Y5 (beam splitter: 660); Pixel size: 2048 × 2048; Scan frequency: 400 Hz; Pinhole: 1 Airy Unit.

EGFP, TdTomato, Hoechst 33342, and TO‐PRO™‐3 or EM48 signals were respectively detected using FITC, RHOD, Dapi, and Y5 filters. HyD detector was used in combination with Y5 filter, while FITC, RHOD, and Dapi filtered signals were detected with PMT detector. To get 40X images, a 2X zoom factor and 20× objective were used.

### 2.7 Image processing

For each mouse, two brain sections were incubated with TO‐PRO™‐3 and two with EM48. At the microscope, the presence of the anterior part of the anterior commissure (aco) within the striatum was verified in order to ascertain the right bregma position (0.145 mm to 0.845 mm). The left or the right striatum of each slice was virtually divided into four areas (I, II, III, IV), including the dorsal‐medial neostriatum (I), dorsal‐lateral neostriatum (II), the ventral‐lateral neostriatum (III), and the ventral‐medial neostriatum with a partial portion of the nucleus accumbens (sector IV, Figure S10). From each of the four striatal regions, 6 images were taken (total of 24 images) and at least four slices for each mouse were imaged. Signals of TdTomato, EGFP and TO‐PRO™‐3 or EM48 were acquired through a multichannel image.

Specifically, TO‐PRO™‐3 and EM48 data were obtained analyzing 4 images for each striatal region and each mouse. Images were processed through ImageJ (v2.1.0).

From each multichannel image, after adjusting brightness and contrast, cells positive either for TdTomato, EGFP and TO‐PRO™‐3 were manually counted. For each striatal region, average of the values of TdTomato, EGFP or TO‐PRO™‐3 was calculated. Then, ratios of TdTomato over TO‐PRO™‐3 and of EGFP over TO‐PRO™‐3 values were determined. Average and standard deviation were calculated across biological replicates.

In striatal slices stained with EM48, we quantified the number of cells co‐expressing EGFP and EM48, as well as TdTomato and EM48. From these counts, we calculated the ratio of EM48‐positive cells within each fluorescently labeled MSN population (i.e., EM48^+^/EGFP^+^ and EM48^+^/TdTomato^+^). These ratios were averaged across biological replicates, with standard deviations reported. By distinguishing between diffuse and aggregated mutant huntingtin using EM48 staining, we were able to determine the percentage of MSNs containing total mutant huntingtin (diffuse or aggregated) and those with aggregated inclusions only.

The process of counting was checked in blinded condition to prevent counting bias by a second operator uninformed of animal genotype and striatal area of the image.

The number of biological replicates for each analysis is reported in the following Table 2:

**TABLE 2:** Number of animals used in the pattern analysis.

*Htt* ^*Q20*^ triple positive	4 months	6 months	>16 months
MSNs counting	7	5	6
*Htt* ^*Q175*^ triple positive	4 months	6 months	>16 months
MSNs counting	9	7	8
Total EM48 counting	6	4	5
Aggr. EM48 counting	5	3	5

*Note*: Table reports the number of *Htt* ^*Q20*^ or *Ht* *t* ^*Q175*^ triple positive mice used for MSNs counting, and total or aggregated EM48 counting.

### 2.8 Brain dissociation into single‐cells suspension

*Drd1‐*TdTomato/*Drd2*‐EGFP/*Htt* ^*Q175*^ or *Drd1‐*TdTomato/*Drd2*‐EGFP/*Htt* ^*Q20*^ triple‐positive mice at 8 weeks of age were chosen for the brain dissociation into single‐cells suspension. After CO_2_ infusion and cervical dislocation, craniotomy was performed and the entire striatum (dorsal and ventral) was dissected and cut into four pieces that were washed in D‐PBS 1X supplemented with calcium, magnesium, glucose, and pyruvate (Gibco, #14287072). Then, dissociation was performed using Adult Brain Dissociation Kit (mouse and rat, Miltenyi, #130‐107‐677), following manufacturer's instructions. Briefly, striatal samples placed into gentleMACS™ C Tubes (Miltenyi #130093237) were mixed with dissociation enzymes and homogenized using 37C_ABDK_02 gentleMACS program on GentleMACS™ Octo Dissociator (Miltenyi, #130095937). Homogenized samples were then filtered using MACS SmartStrainers (70 μm, Miltenyi, #130098426), followed by the debris removal protocol from the Adult Brain Dissociation Kit. Debris Removal Solution was mixed with homogenized samples and, after centrifugation, a density gradient allowed the separation of the sample from debris. Finally, samples were resuspended into 700 μL of sorting buffer, composed of PBS without calcium and magnesium, 2.5% FBS, 0.4% glucose, DNAse I 10 μ/mL, Pen/Strep 1X and 2% B27. Viability of the cells coming from double positive mice was tested adding to the sorting buffer 3 μM DRAQ7 viability marker (ThermoFisher, #D15105). Samples were finally sorted by FACS into pools (of 20–50 neurons for RNA‐seq, and of 200 neurons for validation) or single cells, directly into a 96‐multi‐well plate.

### 2.9 FACS sorting of single‐cell suspension

Preceding the definitive FACS‐sorting, different FACS analysis trials were performed. Cells from negative, TdTomato or EGFP single‐positive or TdTomato and EGFP double positive mice were acquired. Despite the configuration of the instrument allowing a clear separation of EGFP and TdTomato fluorescence, a slight overlap of the two signals was still detectable; thus, electronic signal subtraction was performed.

Forward‐scattered area (FSC‐A) versus side‐scattered area (SSC‐A) light dot plots characterized cells based on their morphology, especially discerning debris signal and myelin fragments. On the other hand, forward‐scattered width (FSC‐W) versus FSC‐A light dot plot was exploited to exclude doublets. Thus, a gate was set to discriminate single cells. Then, viability was tested through DRAQ7 (ThermoFisher, #D15105) to populate another gate. Approximately 70% of cells were alive during FACS sorting in every experiment. Next, FACS sorting was performed to further isolate TdTomato from the EGFP positive cells at single‐cell level. To that end, a nozzle of 100 μm was used and a pressure of 20 psi was exploited. This choice relied on the purpose of reducing possible damage to neurons during the sorting process. Finally, cells were sorted in single‐cell mode, thus utilizing a mask allowing the sorting of only drops free of contaminating particles. The parameters applied during trial analyses were then consistently utilized in all the definitive FACS sorting experiments.

Cells sorting was performed using a BD FACS Aria III, equipped with a blue‐laser (488 nm) and a yellow/green laser (561 nm), thanks to the support of Dr. Isabella Pesce and Alessandro Matté from Cell Analysis and Separation Core Facility at CIBIO.

### 2.10 RNAseq, computational data analysis and selection of candidate genes for target validation

9 pools (5 from Q20 and 4 from Q175) of 20–50 neurons and independent single cells expressing either D1R or D2R coming from entire striata (dorsal and ventral) of two *Htt* ^*Q175*^ and two *Htt* ^*Q20*^ triple‐positive mice at 8 weeks of age were sorted directly into a plate containing 2.5 μL of RLT Plus Lysis Buffer (QIAGEN, #160022859). Genome and transcriptome of individual single cells and pools were separated according to the G&T‐seq [40] workflow. After separation of RNA using Biotin‐SMARTer Oligo dT‐VN beads, reverse transcription into cDNA and library preparation using Illumina Nextera XT technology, cDNA libraries were sequenced using Illumina sequencing technology (Hiseq2000). After sequencing and trimming (TrimGalore‐0.6.6), we checked the quality of RNA‐seq data (FastQC v.0.11.9). Reads were aligned on GRCm39. Genes were considered as expressed if at least 5 counts in 2 or more samples were detected. After having aligned FASTQ files (STAR v.2.5.2b) and generated gene counts, DESeq2 analysis was performed, aiming at dissecting gene expression differences across the following comparisons: D1R (Q175 vs. Q20); D2R (Q175 vs. Q20); Q175 (D1R vs. D2R); Q20 (D1R vs. D2R).

This analysis resulted in lists of differentially expressed genes (DEGs, padj<0.05 and FC >|0.5|) for these comparisons, reporting the fold‐change and their associated adjusted *p*‐values. Then, GO and pathways analysis was performed using clusterProfiler (R package v.4.10.1) in order to discover which biological processes were enriched among the differentially expressed genes in D1R and D2R neurons and comparing *Htt* ^*Q20*^ versus *Htt* ^*Q175*^ conditions. GO terms were then condensed in more general terms by using their semantic similarity (GOSemSim) [41]. Finally, for every *parent* term, the most significant (according to adjusted *p*‐value) *child* GO term was shown in the graphs. Overlap between lists of GO terms was always calculated without semantic similarity application.

Data were further explored to choose interesting candidate DEGs for validation experiments. GO and pathways were manually analyzed to search for most significantly enriched terms and terms related to HD pathology. DEGs—lowest *p*‐values and highest fold change absolute values—involved in the five complexes of oxidative phosphorylation, translation pathways (Mouse Genome Informatics) and well‐established D1R and D2R cell markers were selected for validation by ddPCR using an independent set of triple transgenic animals *N* = 3 (Q20) and 5 (Q175). Results were plotted using GraphPad Prism 8.

2 housekeeping genes (HKGs), *ActB, Tbp* were also included. ddPCR primers for each gene were designed using NIH primer designing tool (https://www.ncbi.nlm.nih.gov/tools/primer-blast/) and PCR Primer Design Tool (Eurofins, https://www.eurofinsgenomics.eu/en/ecom/tools/qpcr-assay-design/). Primers sequences used in this study are listed in Table 3:

**TABLE 3:** Primer sequences used for the targeted validation of the RNA‐seq data.

Gene	FW sequence	RV sequence
*Adora2a*	TGCTTTGTCCTGGTCCTCAC	CATACCCGTCACCAAGCCAT
*Drd1*	CCAGATCGGGCATTTGGAGA	GGTCAATCTCAGTCACTTTTCG
*Pdyn*	CCTTCTGAATCTTGGATCGGC	ACGCAGATCTCAAAGCCTGG
*Penk*	CGACATCAATTTCCTGGCGTG	GTTATCCCAAGGGAACTCGG
*Atp5j2*	GGATAATGATGCGGGATTTCACC	GAGATGCTGCCTTTCCGAAC
*Ndufa4*	AGAAAGAACAACCCAGAGCCA	CAGTTTGCTGTAGTCCACATTC
*Ndufv3*	CTCCGCTCTTGGCATTTGTG	ACTACAGAAACCAGTTCTCCCG
*Rpl38*	GAGATCAAGGACTTTCTGCTGAC	CAGCGAACCTTGAACTTCACATTA
*Uqcc2*	ACGAGAGCTTAGCACGACTG	GCTTGTACTCTTCCACGGACA
*Crym*	TCGGTGCTTCTCTTTGATCCC	AGACACCGCTGCTGTTCTCT
*Sfxn1*	AACGAACAGCTAGAGAATGCGA	TCTCCATAGCTCATTTTCCGTG
*ActB*	GAGTACGATGAGTCCGGCCCCT	ACGCAGCTCAGTAACAGTCCGC
*Tbp*	CAAACCCAGAATTGTTCTCCTT	ATGTGGTCTTCCTGAATCCCT

*Note*: Table reports FW and RV sequences of primers targeting DEGs to be validated by ddPCR.

### 2.11 Lee et al. (2020) dataset analysis

RNAseq data were downloaded from Lee et al. (2020) [18] GEO: GSE152058. Data from 7 D1R and 4 D2R pools of cells, coming from 9 weeks old (wild‐type) WT striata, were specifically analyzed and compared, employing the same analysis pipeline applied on our dataset. Significance of overlap between ours and Lee et al., datasets was calculated using Fisher's exact test exploiting GeneOverlap [42] R package and without semantic similarity application.

### 2.12 Transposable elements expression

Transposable Elements quantification was performed on pools of 20–50 neurons from 8 weeks old triple‐positive animals using TEspeX [43]. TEspeX quantifies TE expression by mapping RNA‐seq data against TE consensus sequences, coding transcripts, and non‐coding transcripts. Only reads mapping with high confidence specifically on TEs were counted, avoiding the inclusion of reads that might be part of exonized TE fragments. RepBase [44] TEs consensus sequences and Ensembl transcript sequences (GRCm39, release‐111) containing all annotated coding and non‐coding transcripts were used as inputs for TEspeX. Differential expression analysis of TEs was then performed using DESeq2 [45].

Experimental condition, cell type and sequencing run were included as covariates in the design formula. Plots were generated using ggplot [46] package in R.

### 2.13 gDNA analysis for CNVs evaluation

Genomic DNA (gDNA) was isolated following G&T‐seq protocol from 8 weeks old triple‐positive animals as previously described. gDNA was subsequently amplified using the PicoPLEX WGA kit, followed by Ampure DNA concentration and purification steps. gDNA libraries were generated using Illumina Nextera XT technology and gDNA libraries were sequenced using Illumina sequencing technology (Hiseq2000). Reads quality was evaluated using FastQC tool (https://www.bioinformatics.babraham.ac.uk/projects/fastqc/) and trimming was accomplished through TrimGalore (https://www.bioinformatics.babraham.ac.uk/projects/trim_galore/). Reads were aligned using STAR (https://github.com/alexdobin/STAR) on GENCODE GRCm39 mouse genome. CNVs were called using SNiPR [47], setting 1 Mb as bins size and MAPD value between 0.1 and 0.65. 3 kmin were required to call a CNV.

### 2.14 Cell death and autophagy evaluation

Levels of markers for cell death (Parp and Casp3) and autophagy (*Lc3b* and Gabarap) were evaluated in the bulk striatum of 6 months old HD animals via WB and/or qPCR, as described below. To compare results with a positive control for cell death, NSC‐34 cell line was treated for 4.5 h with 5 μM staurosporine. To obtain a positive control for autophagy activation, ST*Hdh* Q^7/7^ cell line was treated for 8 h with 400 nM rapamycin, as previously described in Walter C. et al. (2020) [48].

### 2.15 RNA extraction from pool of sorted cells

RNA for targeted validation was extracted from FACS‐sorted pools of 200 cells collected from the total striatum (dorsal and ventral) of 8 weeks old triple positive animals using Norgen Single Cell RNA Purification Kit (#51800) following manufacturer's instructions. Pools of 200 cells were sorted by FACS directly into 96 multi‐well plates containing 100 μL of Buffer RL in each well. Effectiveness of the kit was tested running RT and qPCR on trial samples.

### 2.16 RNA extraction from mouse striata

RNA was extracted from frozen 6 months old mouse total striata (dorsal and ventral) using TRIzol (ThermoFisher) and following manufacturer's instructions. DNase treatment was performed using Zymo‐Spin IC column (Zymo Research) and DNase I (Thermo Scientific #89836).

### 2.17 Retro‐transcription and quantitative PCR

Reverse transcription (RT) was performed on all RNA extracted from pools of cells (10 μL) and on 1 μg of RNA extracted from tissues using SensiFAST™ cDNA Synthesis Kit (Bioline, #BIO‐65054), according to manufacturer's instructions. cDNA was subsequently diluted 1:8 for ddPCR and 1:10 for quantitative PCR (qPCR) using nuclease‐free H_2_O. qPCR was performed using iTaq™ Universal SYBR® Green Supermix (Bio‐Rad, #1725124), following manufacturer's instructions. *Lc3b* was quantified using the following primers: FW: AGCAGCACCCCACCAAGA; RV: GTGGTCAGGCACCAGGAACT [48]. Data was normalized on phosphoglycerate kinase 1 (*Pgk1*) (FW: AAAGTCAGCCATGTGAGCACT; RV: ACTTAGGAGCACAGGAACCAAA) as HKG (ΔCT) and analyzed with the 2^−ΔΔ*Ct*^ method.

### 2.18 Digital droplet PCR

Digital droplet PCR (ddPCR) was chosen to validate DEGs selected from the RNA‐seq data using an independent set of 8 weeks old triple positive transgenic animals (*N* = 3 (Q20) and 5 (Q175)). Primers were resuspended in IDTE 1X TE solution (10 mM Tris, 0.1 mM EDTA) pH 8.0 (Idt, #11‐05‐01‐09) to improve stability of oligos.

The entire procedure was performed using QX200 Droplet Digital PCR Systems (Bio‐Rad). For every sample, a 20 μL master mix containing FW + RV primers (150 nM), QX200™ ddPCR™ EvaGreen Supermix (Bio‐Rad, #1864033) and 10 μL of cDNA was prepared directly in the reaction tube. Each reaction mix was moved into a DG8™ Cartridge (Bio‐Rad, #1864008), closed with DG8™ Gasket (Bio‐Rad, #1863009). Then, samples were nebulized using QX200™ Droplet Generation Oil (Bio‐Rad, #1864005) and the QX200 Droplet Generator (Bio‐Rad, #1864002). Thus, each sample was partitioned into 20,000 droplets. Next, nebulized samples were transferred into a ddPCR™ 96‐Well Plate (Bio‐Rad, #12001925) which was sealed with PCR Plate Heat Seal (Bio‐Rad, #1814040) using the PX1 SEALER (Bio‐Rad, #1814000). The 96‐well plate containing nebulized samples was then incubated in a thermocycler and the PCR protocol for amplification followed, according to manufacturer's instructions. Melting temperature was set at 59°C. Finally, the signal of each droplet was individually detected by the QX200 Droplet Reader (Bio‐Rad, #1864003), allowing for the absolute quantification of the targeted genes. Data analysis was performed using QuantaSoft Analysis Pro Software.

### 2.19 ddPCR and RNAseq data comparison

Gene expression values obtained by ddPCR (copies/μL) and RNA‐seq (CPM) were collected for the following genes: *Actb*, *Drd1*, *Pdyn*, *Penk*, *Adora2a*, *Crym*, *Sfxn1*, *Atp5j2*, *Ndufa4*, *Ndufv3*, *Uqcc2*, and *Rpl38* across the different experimental conditions (Q20‐D1R, Q20‐D2R, Q175‐D1R, and Q175‐D2R). For both techniques, expression values were normalized to *Tbp*, used as HKG, and subsequently log2‐transformed.

To compare ddPCR and RNA‐seq measurements, each log2‐transformed normalized ddPCR value was paired with the corresponding RNA‐seq value obtained for the same gene and experimental condition. Pearson correlation analysis was then performed on the complete set of paired values (*N* = 40 paired data points). Pearson correlation coefficient (*R*) and associated *p*‐value were calculated using a two‐tailed *t*‐test with 38 degrees of freedom (*N* − 2).

### 2.20 Protein extraction and western blot analysis

Proteins were extracted from 6 months old mouse total striata (dorsal and ventral) using RIPA Lysis Buffer (Pierce) and following manufacturer's instructions. Proteins were quantified using BCA Protein Assay kit (Pierce). 30 μg of proteins were run on 4%–12% NuPAGE Bis‐Tris precast gel (Pierce) in MOPS running buffer or on 4%–15% Tris‐Glycine precast gel (Biorad) in Tris/Glycine (Biorad) running buffer. Transfer was performed in Tris/Glycine buffer (BioRad) on Nitrocellulose membrane (Amersham) for 20–45 min at 100 V. Blocking was done in 5% milk in PBS‐Tween. Antibodies [Gapdh (MA515738, 1:2500), Hsp90 (GTX13492, 1:5000), Casp3 (9H19L2, 1:500), Parp (9542 1:1000), Gabarap (ab109364, 1:1000)] were diluted in blocking solution. Images were acquired using ChemiDoc Imaging System (BioRad). Protein expression was calculated by normalizing on HKG (Gapdh or Hsp90) and relative to the average of Q20 striata. Autophagy activity was evaluated by calculating the ratio between cleaved and full‐length Gabarap and Parp cleavage level was evaluated by calculating the ratio between cleaved and full‐length Parp.

### 2.21 CAG typing for somatic instability evaluation by fragment analysis

CAG tract length typing was performed on cDNA obtained from RNA extracted from pools of 200 cell‐sorted D1R‐ and D2R‐MSNs coming from 8 weeks old triple positive mouse total striata (dorsal and ventral), as previously described. 10 replicates for each sample were used as PCR input along with 80 ng weaning ear biopsy DNA as a control. For each cDNA sample replicate, 10.3 μL of cDNA was amplified using Taq PCR Core Kit (QIAGEN, #201223) and FW: FAM–ATGAAGGCCTTCGAGTCCCTCAAGTCCTTC and RV: GGCGGCTGAGGAAGCTGAGGA as primers. PCR protocol was the following: 5 min at 95°C, (30 s at 95°, 30 s at 65°C, 1 min at 72°C) × 35 (DNA from biopsy) or ×40 (cDNA from cells) cycles, 10 min at 72°C. 0.6 μL of PCR product were then mixed with 12 μL of formamide. Samples and 0.3 μL of GeneScan‐600 LIZ Size STD V2.0 (Life Technologies, #4408399) were loaded on SeqStudio Genetic Analyzer Cartridge (#A33671) and run on SeqStudio Genetic Analyzer. Results were analyzed using GeneMapper Software v.6, and only replicates exhibiting clear traces were retained for downstream analysis. Lengths of the highest CAG peak of each “bump” in the genetic analyzer trace were determined and plotted to compare CAG repeat sizes between D1R‐ and D2R‐neurons, providing a qualitative assessment of CAG expansion in each neuronal type. In addition, a somatic instability index and somatic expansion index were quantified as previously described [49] using a 20% threshold cut‐off and adjusting expansion peaks relative to the highest peak in the ear sample (main allele).

### 2.22 Southern blot analyses

The PCR products (as above) and digoxigenin (DIG)‐labeled size marker VIII (Roche) were loaded on 2% agarose gels and electrophoresed in TBE buffer at 60 V for 18 h, then transferred to a positively charged nylon membrane (Roche) by common squash‐blotting technique [50]. The membrane was hybridized with 5 pmol/mL of a 5′ DIG‐labeled (CAG)20 probe (Sigma) in DIG Easy Hybridization Solution (Sigma) overnight at 45°C and then washed twice each with 2X SSC, 0.1% SDS at room temperature for 5 min and 0.1 X SSC, 0.1% SDS at 68°C for 20 min. DIG detection was carried out using the DIG Luminescent Detection system (Sigma) with CPSD substrate according to the manufacturer's instructions (Roche, Cat. No. 11585762001).

### 2.23 Statistical analysis

Experimental data obtained from immunofluorescence, qPCR, western blot, and ddPCR were analyzed and graphed using GraphPad Prism 8. For immunofluorescence analyses, statistical significance was tested using two‐way ANOVA (*α* = 0.05) followed by Tukey's multiple comparison testing, and data are presented as mean ± SD. For qPCR, western blot, and the evaluation of instability and expansion indexes related to somatic instability, two‐tailed unpaired *t*‐tests (*α* = 0.05) were applied. Data are reported as mean ± SD.

To validate targeted findings emerging from our initial RNA‐seq discovery analysis, we used ddPCR. Because these assays were restricted to specific targets and conditions for which a distinct phenotype had already been identified by our sequencing data, we possessed a clear, a priori directional hypothesis. A one‐tailed *t*‐test (*α* = 0.05) was therefore selected as an appropriate statistical test since the direction of the expected difference was strictly defined prior to data collection. Results are shown as mean ± SEM.

For RNA‐seq data, differential expression analysis was performed using DESeq2, which includes internal normalization and adjustment of *p*‐values for multiple testing using the Benjamini–Hochberg method to control the false discovery rate (FDR). In the case of transposable element (TE) expression analysis, TEs with an FDR< 0.1 were considered differentially expressed.

## 3 RESULTS

### 3.1 Transcriptional similarities between striatal D1R‐ and D2R‐ neurons in physiological conditions

To evaluate possible differences between D1R‐ and D2R‐neurons, we generated two knock‐in mouse models carrying 18 (*Htt* ^*Q20*^: “control”) and approximately 190 (*Htt* ^*Q175*^: “HD”) consecutive CAG repeats, each expressing both tdTomato and EGFP fluorescent proteins under the control of *Drd1* and *Drd2* promoters, respectively (Figure S1). A limited, but reliable percentage of cells were double positive for both tdTomato and EGFP, consistent with previous reports [51, 52]. We confirmed the correct neuronal identity of tdTomato and EGFP fluorescent cells by co‐staining with NeuN (pan‐neuronal marker, Figure S1B) and Darpp32 (MSNs marker, Figure S1C). Notably, a relevant number of D1R‐expressing cells, and few D2R‐positive cells, were also observed outside the striatum, particularly in the nucleus accumbens and within the septal area (Figure S1D).

**FIGURE 1:**
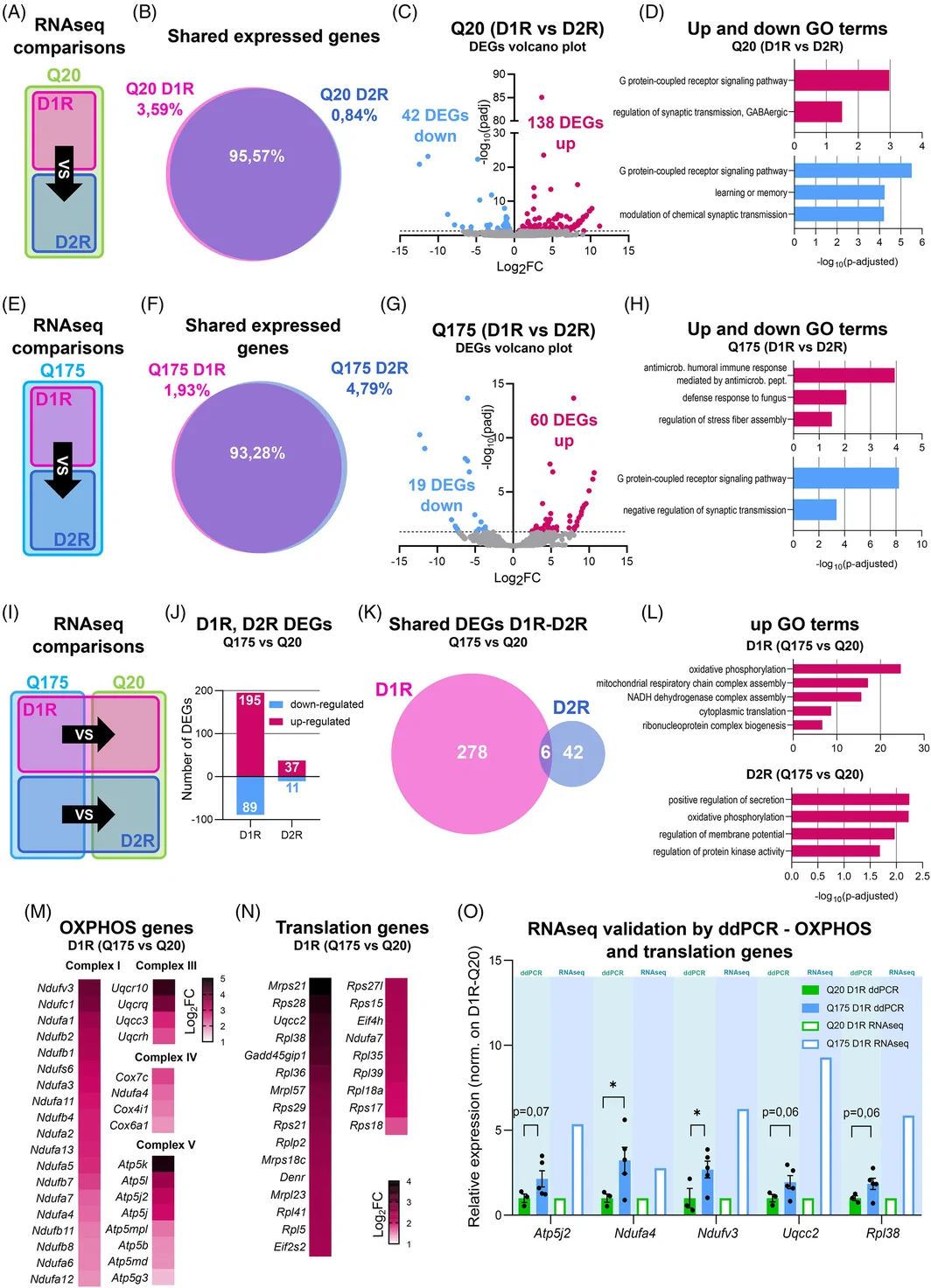
Striatal D1R‐neurons up‐regulate OXPHOS and translation genes expression. Panels (A)–(D) Baseline comparative analysis of D1R and D2R MSNs in the Q20 condition is shown. (A) Experimental scheme of the RNA‐seq workflow. (B) Venn diagram of expressed genes shows a major overlap (95.57%) and a small fraction of unique genes (D1R: 3.59%; D2R: 0.84%). (C) Volcano plot highlights 138 upregulated and 42 downregulated DEGs (D1R vs. D2R). (D) Bar graphs reports representative semantic‐similarity processed GO terms for upregulated (red) and downregulated (blue) DEGs. Panels (E)–(H) Comparative analysis of D1R and D2R neurons in the Q175 condition is presented. (E) Experimental scheme of the RNA‐seq workflow. (F) Venn diagram of expressed genes shows 93.28% overlap, with higher unique expression in D2R (4.79%) than D1R neurons (1.93%). (G) Volcano plot presents 60 upregulated and 19 downregulated DEGs (D1R vs. D2R). (H) Bar graphs reports representative semantic‐similarity processed GO terms for upregulated (red) and downregulated (blue) DEGs. Panels (I)–(N) Disease versus physiological condition comparison (Q175 vs. Q20). (I) Experimental scheme of the cross‐condition comparison is presented. (J) Bar plot shows total DEGs per cell type; D1R neurons exhibited a higher transcriptional response (195 up, 89 down) compared to D2R neurons (37 up, 11 down). (K) Venn diagram depicts minimal overlap (6 shared genes) between D1R and D2R DEGs. (L) Bar graphs shows representative semantic‐similarity processed GO terms for upregulated (red) and downregulated (blue) DEGs, highlighting a strong enrichment for oxidative phosphorylation (OXPHOS) and translation specifically in D1R MSNs. (M) The heatmaps show DEGs detected in our D1R (Q175 vs. Q20) dataset, involved in 4 out of 5 complexes of OXPHOS, all of them being up‐regulated. (N) Heatmap reports the translation‐related genes detected among D1R DEGs, all up‐regulated. (O) The bar graph shows dysregulation of representative genes involved in OXPHOS (*Atp5j2*, *Ndufa4*, *Ndufv3*) and translation (*Uqcc2*, *Rpl38*), validated via digital droplet PCR (ddPCR) using *N* = 3 (Q20) and 5 (Q175) independent triple transgenic mice (biological replicates). Data obtained by ddPCR (green background) and RNAseq (blue background) were compared. Gene expression, obtained as copies/μL for ddPCR and CPM for RNAseq, was normalized on *Tbp* as a HKG. Relative expression was then normalized on D1R‐Q20 samples. All the genes considered were significantly or nearly significantly more expressed in D1R‐Q175 samples in ddPCR, in accordance with RNAseq data. Statistical significance was calculated via one‐tailed *t*‐test (*α* = 0.05). *p< 0.05. Data are represented as mean ± SEM for ddPCR. Statistical and bioinformatics analysis (RNA‐seq): For all the G&T‐seq experiments, 2 mice per genotype (Q20 or Q175) were used. The data presented in the figure were generated starting from 4 pools of FACS sorted Q20 and 5 pools of the Q175 MSNs. Each pool contained a variable number (20–50) of single MSNs. Expressed genes were filtered at >5 CPM in 2 or more samples. DEGs were defined using an adjusted *p*‐value <0.05. GO and pathway analyses were performed using the clusterProfiler R package (v.4.10.1) followed by semantic similarity processing. Statistical significance for GO terms was controlled at a false discovery rate (FDR) using the Benjamini–Hochberg adjustment with a threshold of *p*‐value <0.05.

By applying a mild enzymatic digestion (Miltenyi) and trituration, we isolated 5 pools of 20–50 D1R‐ and D2R‐MSNs from the striatum of 2 control *Htt* ^*Q20*^ animals at 8 weeks of age, and performed Genomic and Transcriptomic sequencing (G&T‐seq [40], Figure 1A). As expected, given the similarities between the cell types, including a shared developmental origin [53], RNAseq profiles revealed a substantial overlap in the genes expressed by D1R and D2R neurons (95.57%, ~13,000 genes). Only a minor fraction of genes was selectively expressed by one of the two populations (D1R only: 3.59%; D2R only: 0.84%—Figure 1B). Accordingly, only 138 genes were identified as significantly more expressed (Table S2) and 42 genes significantly less expressed in D1R‐neurons compared to D2R‐neurons (DEGs, Table S1, Figure 1C). Gene ontology (GO, Table S2) analysis followed by semantic similarity clustering (Table S3) revealed few significant terms, related to “G protein‐coupled receptor signaling pathway”, and “regulation of synaptic transmission” (Figure 1D).

We then conducted a similar, comparative analysis using the dataset from Lee et al. (2020) [18], which employed translating ribosome affinity purification (TRAP) to isolate pools of D1R and D2R neurons from the striata of 9‐week‐old WT mice (Figure S2A). Consistent with our results, the vast majority (88.86%) of genes were expressed by both populations (Figure S2B), while D1R cells exhibit approximately fivefold more unique genes compared to D2R cells (9.51% vs. 1.63%). Although a larger number of DEGs were identified in this dataset (1417 DEGs compared to 180 in our case, Table S4)—possibly due to differences in the cell isolation method—the majority were up‐regulated in D1R cells (741 up vs. 676 down, Figure S2C), in line with our findings. Notably, nearly half of our DEGs in the Q20 (D1R vs. D2R) comparison were also identified as DEGs in the WT (D1R vs. D2R) analysis from Lee et al. (2020) dataset (*p* = 1.61E‐24, Fisher test, Figure S2E), with the vast majority of shared dysregulated genes displaying concordant directional changes (75 out of 77) and similar GO‐term enrichment (Tables S5 and S6), (Figure S2D,F,G; Table S7). Overall, our results, confirmed clear transcriptional similarities between D1R‐ and D2R‐neurons, indicating that under control conditions, no particular transcriptional pathway emerges as possible player on the differential vulnerability observed in response to the HD mutation.

### 3.2 Striatal D1R‐neurons up‐regulate OXPHOS and translation gene expression in response to the HD mutation

To understand how the HD mutation might alter the gene expression profile of MSNs, we collected 4 pools of 40–50 D1R‐ and D2R‐neurons from the striata of 2 *Htt* ^*Q175*^ mice (8‐week‐old), and performed G&T‐seq (Q175‐D1R vs. Q175‐D2R, Figure 1E). 8 weeks of age was selected to model a pre‐symptomatic stage of the disease, characterized by the absence of neuropathology and behavioral phenotypes, but showing some subtle molecular alterations associated with the expanded CAG‐repeat [34]. A substantial percentage of expressed genes was shared between the two cellular populations (93.28%), and the vast majority of these shared genes remained unaltered from the D1R/D2R control condition (Q20, Figure S3). However, in Q175 HD‐mimicking condition, a modestly higher proportion of genes was uniquely expressed in D2R MSNs (4.79%) compared to D1R (1.93%, Figure 1F). We analyzed DEGs (Table S1), identifying 60 up‐regulated genes and 19 down‐regulated genes in D1R compared to D2R‐Q175 MSNs (Figure 1G), enriched for a small number of significant GO terms (Figure 1H, Tables S2 and S3).

To directly dissect the contribution of the HD condition to aberrant transcriptional changes, we compared each neuronal class between healthy and disease conditions [D1R (Q175 vs. Q20) and D2R (Q175 vs. Q20), Figure 1I]. The direct confrontation of D1R‐neurons, Q175 versus Q20, revealed a considerably higher number of DEGs (195 up and 89 down) compared to D2R‐neurons (37 up and 11 down, Table S1), indicating that D1R‐neurons undergo more pronounced changes in response to the expanded CAG‐repeat tract (Figure 1J). Notably, only 6 out of the 320 detected DEGs (up and down) were shared between the two MSN classes (Figure 1K), emphasizing their distinct transcriptional response to the HD mutation.

Interestingly, D1R GO terms (Tables S2 and S3) strongly indicated an up‐regulation of pathways associated with oxidative phosphorylation (OXPHOS) and translation. Although OXPHOS also emerged among dysregulated pathways in D2R neurons, the significant enrichment in D1R neurons (Q175 vs. Q20) was markedly stronger (Figure 1L), with all deregulated genes involved in 4 out of 5 OXPHOS complexes up‐regulated (Figure 1M). Similarly, numerous genes involved in translation were significantly up‐regulated in the D1R comparison (Figure 1N), with the majority (80%) also involved in mitochondrial translation (Figure S4). Prompted by the identification of multiple ribosomal protein‐encoding genes (*Rpl*, *Rps* [54]), we explored whether the more general group of RNA‐binding protein (RBP) genes could be differentially dysregulated in the D1R (Q175 vs. Q20) comparison. Indeed, our analysis highlighted 43 RBPs among the D1R DEGs (Figure S5A), and enriched for GO terms related to “RNA processing and modification,” “ribonucleoprotein biogenesis,” and “transcription” (Figure S5B, Tables S8 and S9).

We then validated some of the described transcriptomic data through digital droplet PCR (ddPCR) on other independent samples of D1R or D2R neurons isolated from 8‐week‐old mice. The neurons were FACS‐sorted into small pools of 200 cells, in line with the protocol preceding RNA‐seq. Relative expression levels were normalized to control condition (Q20‐D1R or Q20‐D2R samples, depending on the analysis) for both RNA‐seq and ddPCR data to facilitate comparative analysis. A pool of well‐established D1R and D2R cellular markers was selected for validation (*Adora2a*, *Drd1*, *Pdyn*, *Penk*, [55, 56, 57, 58]), highly expressed in the specific neuronal subtypes in our dataset. Additionally, we included dysregulated genes involved in OXPHOS (*Atp5j2*, *Ndufa4*, *Ndufv3*) and translation (*Rpl38* and *Uqcc2*).

In line with our RNA‐seq results, D1R (*Drd1*, *Pdyn*) and D2R markers (*Adora2a*, *Penk*) were strongly and specifically expressed (~100 fold) in Q20‐D1R or Q20‐D2R neurons, respectively (Figure S6A). Moreover, both translation and OXPHOS associated genes were up‐regulated in Q175‐D1R cells compared to Q20‐D1R cells (Figure 1O), while no significant differences were detected when comparing Q175‐D2R versus Q20‐D2R cells (Figure S6B), consistent with the RNA‐seq data. Importantly, we observed a linear correlation between ddPCR and RNA‐seq data (Pearson correlation, *R* = 0.87, *p* = 4.21E‐13, Figure S6C).

Finally, to uncover potential differences affecting specific D1R‐ or D2R‐MSN subpopulations [59], transcriptome profiles were also evaluated in individually‐sorted neurons. A principal component analysis (PCA) was conducted, revealing distinct clustering into two groups corresponding to D1R‐ and D2R‐neurons (Figure S7A,B). Subsequently, separate PCA analyses were performed for Q175 and Q20 neurons, and the results were superimposed in Figure S7C,D. The expression of top PC2 markers (*Crym*, *Sfxn1*) was further validated through ddPCR and compared to RNAseq data (Figure S7E).

From the PCA analysis, a clear separation between D1R and D2R neurons into distinct clusters appeared, with D1R (*Drd1*, *Pdyn*) and D2R (*Penk*, *Adora2a*) markers positioned at opposite ends of the PC2 markers list in Figure S7D. Notably, a partial sub‐clustering dependent on the CAG expansion (Q20 or Q175) was observed only in D1R neurons, while D2R neurons remained tightly clustered together. This observation aligns with our earlier findings in pooled samples, where the majority of dysregulated genes were detected in the D1R (Q175 vs. Q20) neuronal subtype.

In summary, within the constraints of a small animal cohort and a limited number of MSNs analyzed, our results suggest active transcriptomic changes evident in D1R‐MSNs already at a pre‐symptomatic stage, involving an up‐regulation of OXPHOS and translation pathways. Conversely, D2R neurons appear to undergo minimal transcriptomic changes in response to the HD mutation at this age.

### 3.3 *Htt* CAG expansion is associated with LINE‐1 elements down‐regulation but not significant copy number variations within MSNs

Given the observations above and the correlation between translation/mitochondrial activity and dysregulation of LINE‐1 (L1) transposable elements (TEs) [60, 61], we investigated possible transcriptional differences in TEs between D1R and D2R neurons and compared control and HD conditions. We analyzed TEs expression at family consensus level using TEspeX. By grouping D1R‐ and D2R‐neurons together and comparing the *Htt* ^*Q20*^ and *Htt* ^*Q175*^ mice, we found that young L1 elements, specifically those belonging to the L1MdTf_I, II, and III and L1MdA_I families, were the most significantly differentially expressed (Figure S8A, Table S10). Additionally, the older L1MdF_II and L1MdF_III families were significantly dysregulated. All L1 elements were downregulated in *Htt* ^*Q175*^ mice compared to *Htt* ^*Q20*^ genotype, providing support to previous observations [28], but extending the findings to striatal MSNs. Specifically, examining the expression variation more closely for the youngest families, we observed the highest expression levels and the most significant differences associated with the L1MdTf_I, II, and III families, which are the youngest group of L1 in mice [62].

While in the mutant mice, young L1 subgroups are reduced in expression, no significant difference could be detected between D1R and D2R neurons. Only a general trend toward lower levels in D1R neurons was noticed, but did not reach full significance in the *Htt* ^*Q175*^ condition (Figure S8B).

Genomic DNA (gDNA) was used to uncover the presence of large copy number variations (CNVs) in pools and single MSNs. FACS‐sorted MSNs from *Htt* ^*Q20*^ and *Htt* ^*Q175*^ 8‐week‐old animals underwent G&T‐seq, CNVs were identified using SNiPR [47], and only aberrations spanning the same chromosomal region and detected in at least 2 samples were taken into consideration. Our analysis revealed sporadic chromosomal ablations and only one chromosomal duplication (one sample, chromosome 4, Figure S9A), with no correspondence with coherent gene‐expression changes, suggesting that the spotted signals were most likely due to background noise. When aggregating the number of aberrations per chromosome or per sample category (Q20‐D1R, Q20‐D2R, Q175‐D1R, Q175‐D2R), we could not discern specific patterns within our samples, correlating with either location or cell type (Figure S9B).

### 3.4 D1R‐MSNs outnumber D2R‐MSNs in HD mice

**FIGURE 2:**
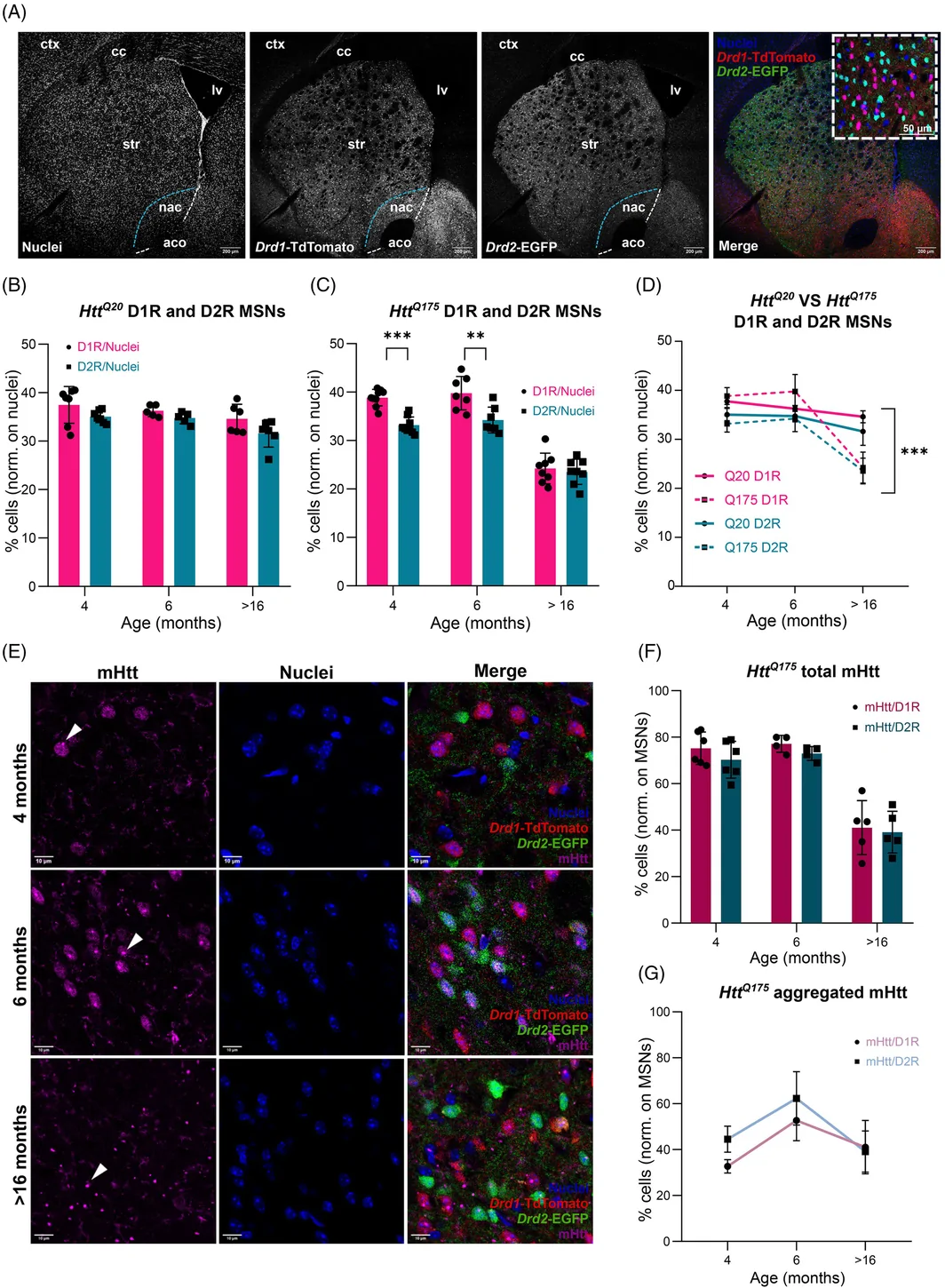
D1R‐MSNs outnumber D2R‐MSNs in *Htt* ^Q175^ mice, while D2R‐MSNs show a greater propensity to accumulate aggregated mutant huntingtin. (A) Representative visual fields of fluorescence imaging experiments, performed on striatal sections from *Htt* ^*Q175*^ and *Htt* ^*Q20*^ mouse models, endogenously expressing Drd1‐TdTomato and Drd2‐EGFP fluorescent proteins. Hoechst 33342 was also used to visualize nuclei. Cortex (ctx), striatum (str), corpus callosum (cc), lateral ventricle (lv), anterior part of the anterior commissure (aco), nucleus accumbens (nac, delimited by blue dashed line) are indicated. The white dashed line outlines the striatal area considered for the analysis. Scale bar: 200 μm. Zoom scale bar: 50 μm. (B) Barplots report the quantification of D1R‐ and D2R‐MSNs from control mice in the entire striatum, at 4 (*N* = 7 mice), 6 (*N* = 5 mice) and >16 (*N* = 6 mice) months of age, highlighting no significant difference. (C) Barplots display D1R‐ and D2R‐MSNs quantification from HD striatum at 4 (*N* = 9 mice), 6 (*N* = 7 mice) and >16 (*N* = 8 mice) months of age. A significantly higher proportion of D1R‐MSNs at 4 and 6 months of age was observed. (D) The graph shows the proportion of D1R‐ (pink) and D2R‐ (blue) MSNs compared between *Htt* ^*Q20*^ (continuous line) and *Htt* ^*Q175*^ (dashed line) mice. At >16 months of age, a strong reduction of both MSNs populations was detected in *Htt* ^*Q175*^ striatum compared to healthy controls. (E) Representative fluorescence images of EM48 antibody, used to detect nuclear mutant huntingtin (purple) within HD mouse striatum MSNs. While at 4 months mainly diffuse nuclear mutant huntingtin is visible, at 6 months more nuclear huntingtin aggregates are observed (arrows), with only aggregates detectable at >16 months. Hoechst 33342 was used to visualize nuclei. Drd1‐TdTomato and Drd2‐EGFP positive cells are visible in merge images. Scale bar: 10 μm. (F) Barplots present total nuclear mutant huntingtin (diffuse and aggregated) within D1R‐ and D2R‐MSNs, calculated in the entire *Htt* ^*Q175*^ striatum. No significant differences were detected at all time points considered (4 months *N* = 6 mice, 6 months *N* = 4 mice, >16 months *N* = 5 mice). A strong decrease of mutant huntingtin within both MSNs populations was observed at the last time point considered, compared to previous time points. (G) The graph shows the quantification of aggregated nuclear mutant huntingtin, analyzed in MSNs in the total HD striatum. At >16 months, approximately 40% of cells exhibit clear nuclear aggregates, while the remaining 60% show no detectable mutant huntingtin signal—neither aggregated nor diffusely distributed. No significant differences were detected at all time points considered (4 months *N* = 5 mice, 6 months *N* = 3 mice, >16 months *N* = 5 mice). From each of the four striatal regions, at least 4 images were taken and analyzed (total of 16 images). Proportion of MSNs was calculated by dividing the number of D1R‐ or D2R‐MSNs by the total number of nuclei. The accumulation of nuclear mutant huntingtin within MSNs was calculated by dividing the number of D1R‐ or D2R‐MSNs positive for EM48 by the total number of D1R‐ or D2R‐MSNs. Statistical analysis was performed applying two‐way ANOVA (*ɑ* = 0.05) followed by Tukey's multiple comparison testing. Data are shown as mean ± SD. ***p< 0.001, **p< 0.01.

To comprehensively address possible differences between D1R and D2R‐MSNs, we performed a spatial quantitative analysis to examine the distribution of these two neuronal subclasses within the mouse striatum (Figure 2A), capitalizing on the expression of fluorescent markers driven by the two dopamine receptors promoters. Fluorescence analysis was carried out at 4, 6, and over 16 months of age, corresponding to early‐symptomatic, symptomatic, and later stages of disease, respectively [34]. We intentionally extended the analysis to later time points compared to the ones studied for G&T‐seq, to grasp possible cellular alterations after the initially detected transcriptional changes, since more pronounced cellular phenotypes might emerge at symptomatic stages.

In control animals, and across the three time points, no significant numerical differences were observed between the two cell types (Figure 2B). However, a significant predominance of D1R‐MSNs over D2R cells was evident at 4 and 6 months in the *Htt* ^*Q175*^ mice (Figure 2C). As the pathologic process progressed, a substantial decrease in both populations of neurons became evident by directly comparing *Htt* ^*Q20*^ and *Htt* ^*Q175*^ animals (Figure 2D), also in line with previous reports [12, 13]. To gain further insight into the distribution of MSNs, we subdivided the striatum into four areas: I, II, III (including the dorsal‐medial, dorsal‐lateral, and ventral‐lateral neostriatum, respectively) and IV (including ventral‐medial neostriatum and a portion of the nucleus accumbens, Figure S10). We observed a modestly greater (though not statistically significant) number of D1R‐MSNs, particularly in the IV sector, even in the control mice (Figure S11A–C), possibly due to the unequal distribution of D1R in the ventral‐medial neostriatum [63]. In *Htt* ^*Q175*^ mice, the outnumber of D1R neurons was evident and significant throughout the striatum at 4 months of age (Figure S11D), but weakened at later disease stages (Figure S11E,F).

### 3.5 D2R‐MSNs exhibit an increased propensity for nuclear accumulation of mutant huntingtin aggregates at early stages

Since mutant huntingtin can form nuclear aggregates that may contribute to neuronal death, we performed anti‐huntingtin EM48 antibody staining to reveal the progressive aggregation of mutant huntingtin within MSNs subpopulations. While both diffuse and aggregated nuclear mutant huntingtin were detectable at earlier disease time points (4 and 6 months), inclusions were only appearing at advanced stages (Figure 2E), consistent with a previous characterization of the *Htt* ^*Q175*^ mouse model [64]. While considering the signal from both aggregated and diffuse forms, no preferential accumulation of mutant huntingtin within D1R or D2R‐MSNs was observed (Figure 2F, S11G,I); instead a clearer phenotype was noticed by focusing on the aggregated form of huntingtin (Figure 2G). In fact, an increased propensity for greater accumulation of aggregated huntingtin was observed in D2R‐neurons as compared to D1R‐MSNs, starting at the earliest time point, as shown by the separate analysis of the four striatal areas (Figure S11J–L).

To better characterize whether the presence of mutant huntingtin aggregates correlates with neurodegeneration (within the mouse striatum), we explored apoptosis and autophagy markers. However, protein lysates from striatum of *Htt* ^*Q20*^ and *Htt* ^*Q175*^ mice (6 months old) showed no overt differences in cleaved caspase3 and Parp (Figure S12A) or the autophagy‐specific markers *Lc3b* and Gabarap (Figure S12B,C).

Although an HD‐specific effect on *Drd1* and *Drd2* promoter efficiency and/or reporter expression levels cannot be completely ruled out, this spatial analysis suggested a higher number of D1R‐MSNs compared to D2R‐MSNs at early disease stages, with both populations declining at advanced time‐points. Additionally, aggregated mutant huntingtin, as detected by EM48 labeling, tended to be preferentially localized to D2R‐MSNs at early stages.

### 3.6 Somatic instability as a potential contributor to the under‐representation of D2R‐MSNs

**FIGURE 3:**
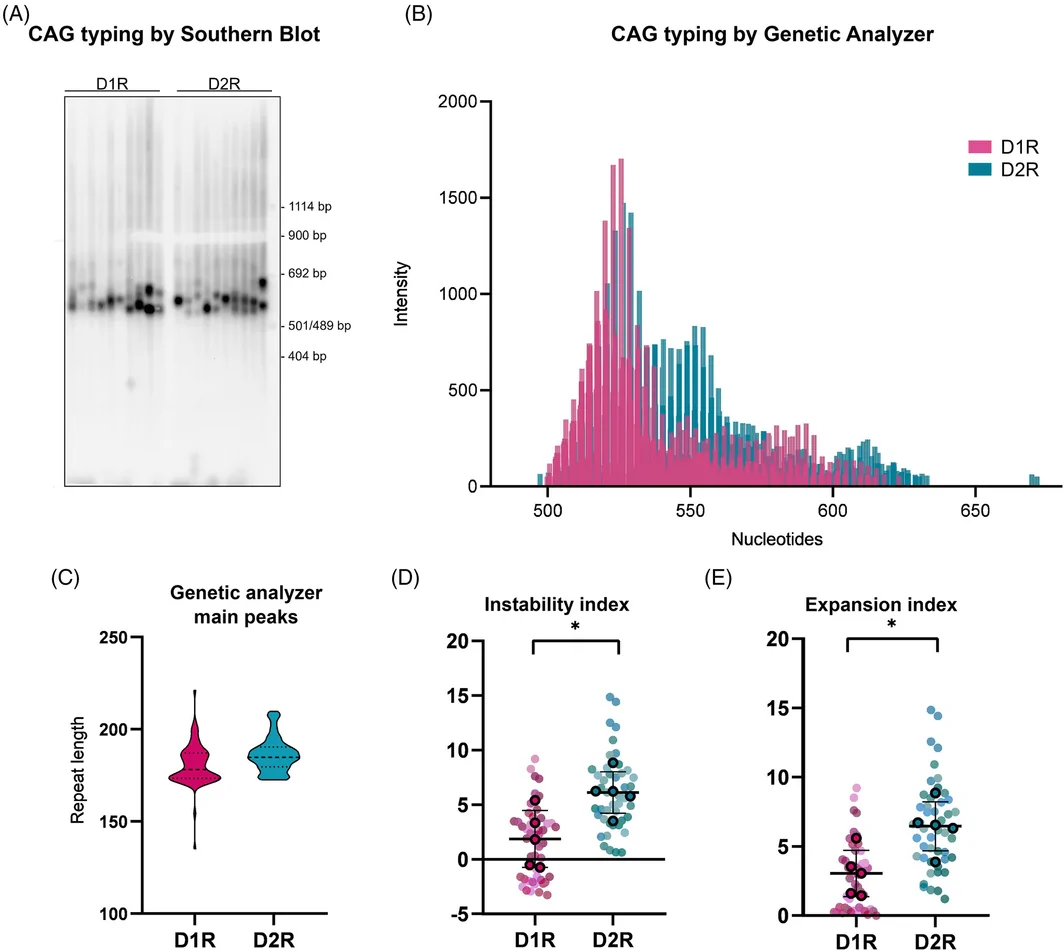
Increased CAG somatic expansion in D2R‐MSNs. (A) Representative southern blot of CAG repeat length in D1R‐ and D2R‐neuron replicates from a single *Htt* ^*Q175*^ mouse. No substantial differences in CAG repeat length were observed between the two cell types. Molecular weights of DNA ladder bands are indicated on the right. (B) GeneMapper profile of Htt CAG repeat distribution across all replicates, illustrating a shift toward longer CAG repeats in D2R‐neurons. (C) Violin plot presenting the highest peaks, determined from the genetic analyzer profiles, supports the trend of greater somatic expansion in D2R‐neurons. Quantification of the CAG Instability Index (D) and CAG Expansion Index (E) indicates a statistically significant increase in somatic expansion in D2R‐neurons compared to D1R‐neurons. CAG typing was performed using small‐pool PCR amplification on cDNA from 200 D1R‐ and D2R‐neurons isolated from *N* = 5 8‐week‐old *Htt* ^*Q175*^ mice, with 10 replicates analyzed per cell type for each mouse. Expansion and instability index are represented as SuperPlots [66], with averages from the five animals highlighted in the foreground, and single replicates for each animal in background. Statistical analysis was conducted with a two‐tailed *t*‐test (*α* = 0.05). Data are shown as mean ± SD. *p< 0.05.

Recent reports suggest a crucial role of somatic instability in neuronal vulnerability to HD [19, 20, 25, 26, 27]. We therefore sought to examine CAG somatic instability in striatal *Htt* ^*Q175*^ D1R‐ and D2R‐neurons (200 cells pools) at 8‐weeks, following a previously established protocol [49, 65]. The *Htt* ^Q175^ CAG repeat was PCR amplified from cDNA of each sample. While southern blot analyses of the PCR products did not reveal notable differences in repeat length distributions between D1R‐ and D2R‐neurons (Figure 3A), fragment analysis, which offers greater resolution of repeat lengths, revealed a profile shift toward longer CAGs in D2R‐neurons (Figure 3B). Given the low number of input cDNA molecules, the PCR products do not form a smooth allele length distribution as typically observed when amplifying bulk genomic DNA, but rather are resolved into several discrete “bumps.” A qualitative comparison of the sizes of each of these bumps supports the trend to greater CAG lengths in D2R‐neurons (Figure 3C). Quantification of the instability index and expansion index [49] revealed significantly higher values in D2R‐MSNs compared to D1R cells (Figure 3D,E). Although our analysis includes only a limited number of neurons from five biological replicates and may therefore not be fully representative of the entire striatum, these findings suggest an increased susceptibility of D2R‐MSNs to CAG tract somatic instability—potentially contributing to their greater vulnerability to disease.

## 4 DISCUSSION

While the selective vulnerability of certain neuronal subtypes is a defining feature of many neurodegenerative diseases, with different neuronal populations uniquely vulnerable in a specific neuro‐dysfunctional context, the underlying molecular mechanisms, both at cell‐autonomous and non‐cell‐autonomous levels, still remain mostly unexplored. Here, we focused on Huntington's disease and MSNs, GABAergic projecting neurons constituting 95% of the striatal neuronal population, and especially vulnerable to the HD mutation [6, 7, 8]. Specifically, among the two subpopulations, D2R‐MSNs, which are involved in inhibiting voluntary movement, start to degenerate earlier [12] compared to the D1R‐MSNs; however, the reasons underlying this differential vulnerability are still to be discovered.

Recent advances in methodologies—such as single‐cell transcriptomics—are providing new angles of investigation into this topic [67]. Thus, here, we resorted to a triple‐positive transgenic HD mouse model, *Drd1*TdTomato/*Drd2*EGFP/*Htt* ^*Q175*^ and control mice, *Drd1*TdTomato/*Drd2*EGFP/*Htt* ^*Q20*^ to explore the molecular and cellular differences of the two types of MSNs at single‐cell level, and compared them under disease and normal conditions using a comprehensive and multi‐faceted approach.

We focused on an early time‐point—8 weeks of age in the mouse—to detect molecular and cellular differences between the two MSN populations at a pre‐symptomatic stage, where no signs of neuropathology or behavioral abnormalities can yet be detected [34]. Indeed, any difference that could be identified before the disease manifestation might represent a valuable target for intervention before symptoms become irreversible. FACS‐sorted TdTomato and EGFP positive cells from dissected striata of HD and control mice were processed through G&T‐seq, allowing simultaneous transcriptomic and genomic analysis from the same cell pools of D1R‐ and D2R‐MSNs. To our knowledge, this approach has never been applied before in the context of HD.

According to the limited differences between D1R and D2R‐MSNs [14, 68], their cooperative roles within movement control [1] and their shared developmental origin [53], we observed 96% of common expressed genes between the two cellular populations in the control condition, indicating significant transcriptional overlap. We found a limited number of DEGs between D1R‐ and D2R‐MSNs—mostly reflecting differential expression of typical markers of the two MSN populations [55, 56, 57, 58]—but we did not identify any clear transcriptional signature or pathway that may explain the selective neuronal susceptibility of D2R neurons. Importantly, these conclusions are fully in line with an independent available dataset published by Lee et al. in 2020 [18].

Many of the transcriptional similarities between D1R‐ and D2R‐neurons were also preserved in the HD condition (93% of shared expressed genes). However, when we directly compared the two cellular populations between Q175 and Q20 controls, we could uncover transcriptional differences of the two neuronal subpopulations with a much more substantial transcriptomic change in the D1R neuronal population in response to the HD mutation, evident already at this pre‐symptomatic stage. This observation was further confirmed by single‐cell data, which revealed distinct clustering of Q20‐ and Q175‐derived D1R cells, indicative of pronounced genotype‐dependent transcriptional alterations. In contrast, control and mutant D2R‐MSNs clustered together, exhibiting minimal transcriptomic divergence. Notably, these findings were further validated by ddPCR on an independent set of triple transgenic animals. GO analysis on D1R‐specific DEGs revealed that the majority of terms associated with top up‐regulated genes were strongly related to oxidative phosphorylation and translation. Specifically, 18% of the up‐regulated genes were involved in 4 out of 5 complexes of oxidative phosphorylation, while 13% of the genes were implicated in the translation pathway (with 80% of these also part of mitochondrial translation). While we used a focused sample size of neuronal pools, our data demonstrate high technical stability with strong inter‐run correlation, low animal‐to‐animal variability and batch effect during clustering, further confirmed by the ddPCR independent approach.

Neurons are particularly sensitive to ATP levels, which are mainly provided through the OXPHOS pathway [69, 70]. Dysfunctions in mitochondrial activity have been described in several neurodegenerative diseases [71], and specifically in HD, with alterations in various aspects, such as mitochondrial biogenesis, oxidative stress, fusion and fission, trafficking, and calcium homeostasis [72, 73, 74]. Our findings describe an up‐regulation of the ATP production pathways in D1R‐neurons, already at pre‐symptomatic HD stages. This observation suggests the activation of an early compensatory mechanism, possibly contributing to D1R‐MSNs initial resilience to HD neurodegeneration. Supporting this hypothesis, recent studies described that ATP supplementation in a mouse model of Parkinson's Disease could mitigate pathological phenotypes [75]. Similarly, HD patients—in an open‐label trial—treated with drugs increasing brain ATP levels exhibited a temporary corrected brain energy profile [76].

In contrast to previous studies, here we provide a transcriptional snapshot of an early, pre‐symptomatic disease stage, rather than focusing on later time points, when multiple and interconnected dysfunctional pathways might be activated. Similarly to our findings, a study on 15 weeks old R6/1 and R6/2 HD mouse models also reported increased OXPHOS and translation pathways in the striatal transcriptome [77].

Differently, Lee et al. (2020) [18] reported reduced OXPHOS activity and mitochondrial damage in D2R‐MSNs from 6‐month‐old *Htt* ^*Q175*^ mice, leading to activation of the interferon pathway. Although we did not detect differential expression of interferon‐related genes, the up‐regulation of OXPHOS genes in D1R‐MSNs that we detected at the pre‐symptomatic stage likely reflects the same pathological process from a different perspective—Lee et al. observed OXPHOS down‐regulation in D2R‐MSNs, whereas we identify a possibly compensatory up‐regulation in D1R‐MSNs. Collectively, these findings support an early, cell‐type–specific inability of D2R‐MSNs to maintain mitochondrial function.

Genomic variability can arise from LINE‐1 retro‐transposition [78, 79, 80], the most abundant class of transposable elements in humans [81]. By inducing DNA double‐strand breaks and genomic instability, LINE‐1 activity influences brain physiology, somatic mosaicism, and disease susceptibility. Aberrant LINE‐1 activation has been linked to neurodevelopmental defects, neuroinflammation, and neurodegeneration [82]. Notably, dysregulated LINE‐1 expression has been correlated with alterations in mitochondrial protein translation [60, 61]. In HD, studies of *post‐mortem* tissue and *Htt* ^*Q175*^ mouse brains have shown reduced LINE‐1 expression in the striatum, inversely correlating with CAG repeat length [28]. Here, we examined whether LINE‐1 expression differs between D1R‐ and D2R‐MSNs. LINE‐1 and young LINE‐1 elements were overall down‐regulated in HD MSNs but showed no significant differences between the two populations, suggesting that retro‐transposition does not directly contribute to MSN vulnerability. Similarly, gDNA analysis from pooled and individual D1R‐ and D2R‐MSNs revealed no validated large CNVs. Despite technical limitations, these findings align with reports that structural variants in the adult mouse brain are rare, typically small and developmentally confined. [83].

Moving from the molecular characterization to the distributional and cellular perspective, we then analyzed the distribution of D1R‐ and D2R‐MSNs within the mouse striatum at various time points—all more advanced compared to when the molecular analysis was conducted—early symptomatic (4 and 6 months of age) and at advanced stages of the disease (>16 months of age). This analysis covered the entire neostriatum as well as specific striatal sub‐areas (named I, II, III, and IV).

In control conditions, the proportion of D1R‐ and D2R‐MSNs across the entire striatum or within individual subregions was not majorly different. However, in Q175 mutant mice, a numerical predominance of D1R‐MSNs at 4 and 6 months of age became evident. This characteristic faded away at the advanced disease stage, where both neuronal populations markedly declined. This data supports a better ability of D1R to cope with the HD mutation, maintaining their sparseness longer compared to D2R. Notably, while our imaging analysis relied on EGFP and tdTomato reporter fluorescence to determine MSN subclass identity—and a genotype‐specific effect on promoter efficiency cannot be entirely ruled out—comparable *Drd1/Drd2* transcript levels and stable reporter fluorescence intensities across genotypes (data not shown) indicate this is not a major confounding factor.

Targeted analysis of mutant huntingtin inclusions within MSNs revealed a trend toward larger accumulation of aggregated huntingtin in D2R‐MSNs, particularly within the dorsomedial neostriatum. Given that mutant huntingtin aggregates might be toxic [84, 85, 86], then, D2R‐MSNs could be the first to undergo degeneration. This conclusion aligns well with existing literature indicating that D2R‐MSNs are affected earlier in HD, while both populations are degenerating at advanced stages [12, 13]. Furthermore, it also fits with existing reports from HD patients, describing striatal degeneration which starts from the dorsal‐medial area [87, 88]. However, when we explicitly searched for markers of cell death, specifically looking for apoptosis and autophagy, no significant difference between HD and control animals could be detected at 6 months of age. While these markers might peak in their expression at later time points (>16 months), concomitant to the observed MSNs loss, individual MSNs might reach the “final catastrophic stage” asynchronously [19], thus preventing a detectable activation of autophagy or apoptotic pathway in bulk striatum. While interesting, our neuropathologic analysis is limited and would benefit from an investigation of neuronal atrophy versus loss, and synaptic and/or dendritic alterations. Interestingly, studies in yeast reported that higher ATP production reduces cytoplasmic viscosity, affecting liquid–liquid phase separation and enhancing molecular solubility [89]. Liquid–liquid phase separation contributes to aggregate formation in several neurodegenerative diseases [90, 91], including HD [92]. Thus, it is tempting to speculate that the increased OXPHOS observed in D1R‐neurons may reduce mutant huntingtin aggregates formation, possibly contributing to their resilience to the HD pathology.

Emerging evidence indicates that CAG somatic expansion, which can be dramatically extensive in patients' MSNs (reaching >500 repeats), is both necessary and sufficient to trigger HD. When the CAG tract exceeds 150 repeats, a significant dysregulation of gene expression is observed, accompanied by a dramatic loss of MSNs [19]. Here, we employed our triple transgenic mouse model to compare CAG somatic instability in D1R‐ and D2R‐neurons. Although our results suffer from some technical limitations—a population of D1R‐positive neurons was detected in the nucleus accumbens, particularly within the septal region [93], suggesting a possible, though limited, contribution from septal D1R‐expressing interneurons to the somatic instability analysis and a limited number of animals was used, possibly not fully reflecting the diversity of CAG‐expanded *Htt* variants—our data suggest a modestly higher somatic expansion in D2R‐MSNs with limited transcriptomic alterations, detected as early as 8 weeks of age.

Our observations suggest a correlation between somatic *Htt* instability and mutant Htt aggregates deposition. On the other hand, the lack of somatic mosaicism—at least at the level of large CNVs—coupled with pronounced *Htt* CAG somatic mosaicism—supports that the latter phenotype is not related to a general genomic instability mechanism, but rather driven by the CAG‐repeat length itself. While these observations are still correlative, these points warrant a deeper investigation and understanding.

Overall, our multidimensional and integrated study highlighted specific molecular mechanisms differentially altered in the two MSNs populations from very early time points on, at presymptomatic stages. D1R‐MSNs showed higher OXPHOS rate and translation which might correlate with reduced mutant huntingtin aggregates and their resilience to HD.

On the other hand, these characteristics might also suggest a distinct “neurodevelopmental trajectory” for D1R‐MSNs in response to the HD mutation: thus, the contribution of mutant huntingtin to developmental pathways might act as sensitizer or protector to later degenerative processes [94, 95, 96, 97].

In conclusion, deeper molecular efforts to characterize differences in MSNs sub‐populations already early in development and at single‐cell level are needed to fully capture the intricate complexity of the altered molecular and cellular pathways and further capitalize on this knowledge to conceive novel therapeutic strategies.

## AUTHOR CONTRIBUTIONS

M.B. conceived, supervised the study and acquired funds. V.C.W. and E.D. contributed to study supervision. G.B. designed and performed experiments, analyzed data, prepared figures. M.P., A.S., E.D., M.T., and V.P., performed RNA‐seq data analysis. M.L., S.K.T., E.O., V.C.W., contributed to somatic instability evaluation. S.G., J.G., and T.V. performed G&T sequencing. L.G. and R.S. identified and analyzed LINE elements expression. D.F. contributed to model characterization and spatial analysis. A.R. analyzed gDNA data. R.M. provided *Drd1*‐TdTomato and *Drd2*‐EGFP transgenic models. T.T. and I.P. contributed to cell MSNs FACS sorting protocol setting. S.V. and J.M. assisted with revisions. J.M. contributed to critical discussion and proof‐reading of the manuscript. G.B. wrote the paper together with M.B. All authors read and approved the final version of the manuscript.

## FUNDING INFORMATION

This work was supported by the University of Trento. M.B. was a recipient of a Marie Skłodowska Curie reintegration fellowship (the European Union's Horizon 2020 Research and Innovation Program, GA n 706567). Dept. CIBIO Core Facilities are supported by the European Regional Development Fund (ERDF) 2014–2020 and 2021–2027. This work has received funding from the Italian Ministry of University and Research (MUR) under the initiative “Dipartimenti di Eccellenza 2023‐2027 (Legge 232/2016)”—Dept. CIBIO. V.C.W was funded by NIH grant R01 NS049206.

## CONFLICT OF INTEREST STATEMENT

V.C.W. was a founding scientific advisory board member with a financial interest in TripletTherapeutics Inc. Her financial interests were reviewed and are managed by Massachusetts General Hospital (MGH) and Mass General Brigham (MGB) in accordance with their conflict‐of‐interest policies. V.C.W. is a scientific advisory board member of LoQus23 Therapeutics Ltd. and has provided paid consulting services to Acadia Pharmaceuticals Inc., Alnylam Inc., Biogen Inc., Passage Bio and Rgenta Therapeutics.

## Supporting information


**Supplementary Figure S1:** Characterization of *Drd1‐*TdTomato, *Drd2*‐EGFP mouse model. Representative immunofluorescence images of *Drd1*‐TdTomato, *Drd2*‐EGFP and (A), (D) nuclear (ToPro3), (B) pan‐neuronal (NeuN), and (C) MSNs (Darpp32) markers co‐localization in the mouse striatum. (D) D1R‐expressing cells were also detected outside the striatum, specifically in the nucleus accumbens area. These cells might represent D1R‐expressing interneurons. Str = striatum, nac = nucleus accumbens, aco = anterior part of the anterior commissure, lv = lateral ventricle. Scale bars: 50 μm (A), (B), 20 μm (C), 100 μm (D).
**Supplementary Figure S2:** D1R‐ and D2R‐neurons show similar transcriptional patterns in physiological condition. (A) Gene expression of D1R and D2R neurons from 9 weeks old WT mouse striata from Lee et al., 2020 dataset was analyzed applying the same pipeline used for our dataset. (B) Venn diagrams show expressed genes (at least 5 CPM in 2 or more samples) compared between D1R and D2R cells. **C**. Significant DEGs (adjusted *p*‐value< 0.05) are represented in the volcano plot. 741 up‐regulated and 676 down‐regulated DEGs were detected. (D) Top 10 up‐regulated (red) and down‐regulated (blue) GO terms after semantic similarity processing are reported. (E) Venn diagram displays DEGs detected from Lee et al. dataset compared to the ones observed within our study in Q20 (D1R vs. D2R) condition, with 75 shared genes (*p* = 1.61E‐24, Fisher's exact test). (F) Venn diagram reports down‐regulated GO terms comparing Lee et al. and our results, revealing more than 50% (101 over 177, *p* = 6.8E‐50 Fisher's exact test) of shared terms. (G) The graph describes overlapping GO terms between Lee et al. (WT) and our Q20 dataset, after compacting by semantic similarity. The enrichment level of GO terms was quantified using the GR/BG ratio (gene ratio/background).
**Supplementary Figure S3:** Overlap between expressed genes in D1R‐ and D2R‐neurons in disease and control condition. Venn diagram shows number of genes detected as expressed (at least 5 CPM in 2 or more samples) in the four cellular types (D1R‐Q20, D2R‐Q20, D1R‐Q175, D2R‐Q175).
**Supplementary Figure S4:** D1R dysregulated genes involved in mitochondrial translation. The heatmap represents the 25 mitochondrial translation genes identified among dysregulated genes, contrasting D1R‐Q175 versus D1R‐Q20 condition. All of them are up‐regulated.
**Supplementary Figure S5:** RNA binding proteins (RBPs) are dysregulated in the D1R (Q175 vs. Q20) comparison. (A) The heatmap depicts the 43 RBPs found dysregulated in the comparison between D1R‐Q175 and D1R‐Q20 cells. 38 of them were up‐regulated. (B) GO terms analysis was performed on detected RBPs genes, and the bar graph represents the top 10 GO terms (after semantic similarity processing).
**Supplementary Figure S6:** RNAseq data validation via ddPCR. Dysregulated gene expression detected by RNAseq data was validated by digital droplet PCR (ddPCR) performed on RNA extracted from pools of 200 FACS‐isolated D1R‐ or D2R‐neurons. (A) In the barplot, MSNs markers (*Drd1* and *Pdyn* for D1R, *Penk and Adora2a* for D2R) expression were compared in Q20‐D1R and Q20‐D2R cells. Similarly to RNAseq data (blue background), ddPCR‐detected expression (green background) of *Drd1* and *Pdyn* was significantly higher in D1R neurons compared to D2R, while *Penk* and *Adora2a* were significantly more expressed in D2R cells. (B) Barplot represents OXPHOS (*Atp5j2, Ndufa4, Ndufv3*) and translation (*Uqcc2, Rpl38*) genes expression in Q20‐D2R and Q175‐D2R cells. No significant differences were detected. Gene expression, obtained as copies/μL for ddPCR and CPM for RNAseq, was normalized on *TBP* as HKG. Relative expression was then normalized on D1R‐Q20 samples (A) and on D2R‐Q20 samples (B). Statistical significance was calculated via one‐tailed *t*‐test (*α* = 0.05). For ddPCR, data are represented as mean ± SEM. **p< 0.01, ***p <0.001. *N* = 4 (Q20) and 5 (Q175) biological replicates. (C) The scatter plot represents ddPCR and RNAseq expression data comparison via a correlation analysis. Expression obtained for every gene analyzed (*Actb*, *Drd1*, *Pdyn*, *Penk*, *Adora2a*, *Crym*, *Sfxn1*, *Atp5j2*, *Ndufa4*, *Ndufv3*, *Uqcc2, Rpl38*) in different conditions (Q20‐D1R, Q20‐D2R, Q175‐D1R, Q175‐D2R) was normalized on *TBP* as a housekeeping gene. Data were then represented as Log_2_(normalized expression). A high level of correlation between RNAseq and ddPCR results was obtained (Pearson *R* = 0,87, two‐tailed t‐test p‐value = 4,21E‐13, degrees of freedom: 38).
**Supplementary Figure S7:** PCA on single cells highlights distinct clustering of D1R and D2R‐neurons. (A) The scatterplot represents an unbiased principal component analysis (PCA), which was initially performed on single cells gene expression. D1R‐ (red) and D2R‐ (blue) neurons tend to clusterize based on PC1. (B) PC1 and PC2 marker genes are shown. (C) D1R and D2R sample density, based on PC1 and PC2 from the PCA shown in (D) Scatter plot reports PCA, performed on single cells by overlapping PCAs performed on Q20 and Q175 cells separately. This PCA illustrates separation between D1R (circles) and D2R cells (triangles), with D1R‐neurons showing greater dispersion between Q175 (blue) and Q20 (green) conditions. PC1 explains 7.2% of variance while PC2 explains 4.2% of variance. PCs markers and their relative weights are shown below the PCA. (E) The barplot represents PCA markers *Crym* and *Sfxn1* expression, detected via ddPCR in Q20 and Q175 D1R‐ and D2R‐neurons. Gene expression was comparable (no significant differences) to RNAseq results. *N* = 3 biological replicates. Gene expression, obtained as copies/μL for ddPCR and CPM for RNAseq, was normalized on *TBP* as a housekeeping gene. Relative expression was then normalized on D1R‐Q20 samples. Statistical significance was calculated via one‐tailed *t*‐test (*α* = 0.05). For ddPCR, data are represented as mean ± SEM.
**Supplementary Figure S8:** LINE‐1 transposable elements (TEs) are down‐regulated in Q175 neurons compared to Q20. (A) Volcano plot shows LINE‐1 TEs, and L1MdF_II and L1MdF_III families being down‐regulated in the disease condition compared to control. TEspeX was used on transcriptome data collected from small pools of MSNs to quantify and compare TEs (LINE‐1, Young LINE‐1 and others) expression in Q175 and Q20 neurons, without separating D1R‐ from D2R‐expressing cells. (B) The boxplot shows that expression of young LINE‐1 elements specifically investigated in D1R and D2R neurons in healthy and disease condition, revealing a tendency toward lower levels in D1R for L1MdTf_I, II and III elements. Statistical analysis performed using DESeq2 (padj). *p< 0.1, **p< 0.05, ***p< 0.001.
**Supplementary Figure S9:** MSNs do not present large copy number variations (CNVs). (A) CNVs analysis was performed in pools and single D1R‐ and D2R‐ neurons across the entire genome. Chromosomal regions with aberrations in at least two different samples are represented in the heatmap. A condition of diploidy (normal condition) is represented in white, while chromosomal duplications and ablations are represented respectively in red and blue. On the *Y*‐axis, samples are grouped per type: Q175‐D1R, Q175‐D2R, Q20‐D1R, Q20‐D2R. On the *X*‐axis, chromosomal regions with consecutive chromosomal aberrations are indicated and separated by chromosomal number. Overall, the samples present a condition of diploidy in almost the entire genome. Few chromosomal ablations and one chromosomal duplication were detected across the genome. However, the comparison with RNAseq data indicated that this signal is most likely due to background noise. (B) In the heatmap, the number of chromosomal alterations (both ablations and duplications) were grouped per single chromosome (*Y*‐axis) and per sample type (*X*‐axis). The sum of chromosomal alterations is indicated on the bottom and on the left of the graph. While the majority of aberrations can be ascribable to chromosome 19, no significant differences were detected between different samples, indicating that the presence of CNVs is not related to cell type or the disease condition.
**Supplementary Figure S10:** Immunofluorescence image of a mouse striatal section. Mosaic representative immunofluorescence image of a *Htt* ^*Q20*^ striatal section, positive for *Drd1*‐TdTomato, *Drd2*‐EGFP and Nuclei (ToPro3) stainings. The striatal section was virtually divided into four areas: dorsal‐medial (I), dorsal‐lateral (II), ventral‐lateral (III) neostriatum, and ventral‐medial neostriatum with a portion of the nucleus accumbens (IV). In the figures are indicated the corpus callosum (cc), the striatum (str), the cortex (ctx), the lateral ventricle (lv), nucleus accumbens (nac, delimited by dashed line) and the anterior part of the anterior commissure (aco). Cell counting was performed within the striatum region delimited by the cortex, the lateral ventricle, and the solid line. Scale bar: 200 μm.
**Supplementary Figure S11:** MSNs distribution and mutant huntingtin accumulation within striatal areas. D1R‐ and D2R‐MSNs proportion and mutant huntingtin accumulation within MSNs was evaluated into four striatal areas: dorsal‐medial (I), dorsal‐lateral (II), ventral‐lateral (III) neostriatum, and ventral‐medial neostriatum with a portion of the nucleus accumbens (IV), across different time points (4, 6 and >16 months of age), and represented in bar graphs. (A)–(C) The MSNs proportion at 4, 6 and >16 months of age in *Htt* ^*Q20*^ mice did not reveal any significant differences but only a nearly significant tendency in having more D1R‐ than D2R‐MSNs in region IV. (D)–(F). D1R‐MSNs are instead significantly greater in number compared to D2R in the entire *Htt* ^*Q175*^ striatum at 4 months. At 6 months, this difference is still visible only in the ventral striatum. At >16 months, no more significant differences are observed, but the same tendency is still detectable in region IV. A great decrease of both MSNs populations is present at this time point. MSNs proportion was calculated by normalizing the number of D1R‐ and D2R‐ cells over the total number of cells (nuclei). (G)–(I). The amount of total (diffuse and aggregated) mutant huntingtin within D1R‐ and D2R‐ neurons does not differ across the *Htt* ^*Q175*^ striatum and between D1R‐ and D2R‐MSNs, but at advanced disease stage the proportions of neurons presenting nuclear mutant huntingtin is decreased. (J)–(L) In *Htt* ^*Q175*^ striatum, nuclear aggregated mutant huntingtin is borderline significantly more accumulated within D2R‐MSNs compared to D1R at 4 months of age. This tendency is then lost at 6 and >16 months of age. The accumulation of nuclear mutant huntingtin within MSNs was calculated by dividing the number of D1R‐ or D2R‐MSNs positive for EM48 by the total number of D1R‐ or D2R‐MSNs. Statistical analysis was performed applying two‐way ANOVA (*ɑ* = 0.05) multiple comparison. ***p< 0.001, *p< 0.05. Number of biological replicates for every experiment are indicated in figure. Data are represented as mean ± SD.
**Supplementary Figure S12:** Neurodegeneration or cell death are not detectable in *Htt* ^*Q175*^ 6 months old mouse striatum. (A) The presence of cell death was evaluated in protein extracts from 6 months old *Htt* ^*Q20*^ and *Htt* ^*Q175*^ mouse striata using antibodies for cleaved (CL) Caspase 3, cleaved and full‐length (FL) Parp, as shown in the representative western blot. Positive control for cell death is represented by NSC‐34 cell line treated with Staurosporine. While Caspase 3 signal was completely undetectable from both the mouse genotypes, relative protein level expression of Parp obtained by quantifying WB image and normalizing on Gapdh as HKG did not reveal any significant difference between *Htt* ^*Q20*^ and *Htt* ^*Q175*^ mice. *N* = 4 biological replicates. (B) The barplot represents autophagy marker *Lc3b* expression, evaluated on RNA extracted from 6 months old *Htt* ^*Q20*^ and *Htt* ^*Q175*^ mouse striata. Positive control for autophagy activation is represented by St*Hdh* Q^7/7^ cell line treated with rapamycin. Quantification of the gene expression (2^−ΔΔ*Ct*^ method, normalizing on *Pgk1* as HKG) showed no difference between the two genotypes. *N* = 4 biological replicates (mouse striata), *N* = 2 technical replicates for positive/negative controls. (C) As shown on the left by representative western blot, autophagy levels were also evaluated in protein extracts from 6 months old *Htt* ^*Q20*^ and *Htt* ^*Q175*^ mouse striata using antibodies for CL and FL Gabarap. Hsp90 was used as HKG. On the right, the barplot shows autophagy levels in *Htt* ^*Q20*^ and *Htt* ^*Q175*^ mice, evaluated by calculating the ratio between CL and FL Gabarap. No significant differences were observed. *N* = 4 biological replicates. Statistical analysis was performed by applying two‐tailed *t*‐test (*ɑ* = 0.05) and data are presented as mean ± SD.

**Table S1:** DEGs.

**Table S2:** Significant GO terms.

**Table S3:** GO terms after semantic similarity.

**Table S4:** DEGs D1R versus D2R Lee et al. 2020.

**Table S5:** GO terms Lee et al. 2020.

**Table S6:** Significant GO terms after semantic similarity Lee et al. 2020.

**Table S7:** Shared GO terms Q20 versus WT (Lee et al. 2020).

**Table S8:** RBPs GO terms.

**Table S9:** RBPs GO terms after semantic similarity.

**Table S10:** TEs expression.

## Data Availability

The datasets generated during the current study are accessible at GEO repository with accession numbers: GSE307771 and GSE307772. All data will be available from the corresponding author on reasonable request.
