# Supplementary material for: Decoding neuronal vulnerability: Multidimensional analysis of D1R‐ and D2R‐ medium‐sized spiny neurons in Huntington's disease
Source: Brain Pathol. 2026 Aug 26;36(6):e70135. doi: 10.1111/bpa.70135 (PMC13509169; doi:10.1111/bpa.70135)
Supplement: Supplementary file 1 [file BPA-36-e70135-s007.docx]

**Bergonzoni *et al.,* 2025 – Supplementary figures**

**Supplementary figure S1: characterization of *Drd1-*TdTomato, *Drd2*-EGFP mouse model.** Representative immunofluorescence images of *Drd1*-TdTomato, *Drd2*-EGFP and **(A,D)** nuclear (ToPro3), **(B)** pan-neuronal (NeuN), and **(C)** MSNs (Darpp32) markers co-localization in the mouse striatum. **D.** D1R-expressing cells were also detected outside the striatum, specifically in the nucleus accumbens area. These cells might represent D1R-expressing interneurons. Str = striatum, nac = nucleus accumbens, aco = anterior part of the anterior commissure, lv = lateral ventricle. Scale bars: 50 µm (A,B), 20 µm (C), 100 µm (D).

**Supplementary figure S2: D1R- and D2R-neurons show similar transcriptional patterns in physiological condition. A.** Gene expression of D1R and D2R neurons from 9 weeks old WT mouse striata from Lee *et al.*, 2020 dataset was analysed applying the same pipeline used for our dataset. **B.** Venn diagrams show expressed genes (at least 5 CPM in 2 or more samples) compared between D1R and D2R cells. **C.** Significant DEGs (adjusted p-value<0,05) are represented in the volcano plot. 741 up-regulated and 676 down-regulated DEGs were detected. **D.** Top 10 up-regulated (red) and down-regulated (blue) GO terms after semantic similarity processing are reported. **E**. Venn diagram displays DEGs detected from Lee et al. dataset compared to the ones observed within our study in Q20 (D1R vs D2R) condition, with 75 shared genes (p=1,61E-24, Fisher’s exact test). **F.** Venn diagram reports down-regulated GO terms comparing Lee *et al.* and our results, revealing more than 50% (101 over 177, p=6,8E-50 Fisher’s exact test) of shared terms. **G.** The graph describes overlapping GO terms between Lee *et al.* (WT) and our Q20 dataset, after compacting by semantic similarity. The enrichment level of GO terms was quantified using the GR/BG ratio (gene ratio/background).

**Supplementary figure S3: Overlap between expressed genes in D1R- and D2R-neurons in disease and control condition.** Venn diagram shows number of genes detected as expressed (at least 5 CPM in 2 or more samples) in the four cellular types (D1R-Q20, D2R-Q20, D1R-Q175, D2R-Q175).

**Supplementary figure S4: D1R dysregulated genes involved in mitochondrial translation.** The heatmap represents the 25 mitochondrial translation genes identified among dysregulated genes, contrasting D1R-Q175 vs D1R-Q20 condition. All of them are up-regulated.

**Supplementary figure S5: RNA binding proteins (RBPs) are dysregulated in the D1R (Q175 vs Q20) comparison. A.** The heatmap depicts the 43 RBPs found dysregulated in the comparison between D1R-Q175 and D1R-Q20 cells. 38 of them were up-regulated. **B.** GO terms analysis was performed on detected RBPs genes, and the bar graph represents the top 10 GO terms (after semantic similarity processing).

**Supplementary figure S6: RNAseq data validation via ddPCR.** Dysregulated gene expression detected by RNAseq data was validated by digital droplet PCR (ddPCR) performed on RNA extracted from pools of 200 FACS-isolated D1R- or D2R-neurons. **A.** In the barplot, MSNs markers (*Drd1* and *Pdyn* for D1R, *Penk and Adora2a* for D2R) expression were compared in Q20-D1R and Q20-D2R cells. Similarly to RNAseq data (blue background), ddPCR-detected expression (green background) of *Drd1* and *Pdyn* was significantly higher in D1R neurons compared to D2R, while *Penk* and *Adora2a* were significantly more expressed in D2R cells. **B.** Barplot represents OXPHOS (*Atp5j2, Ndufa4, Ndufv3*) and translation (*Uqcc2, Rpl38*) genes expression in Q20-D2R and Q175-D2R cells. No significant differences were detected. Gene expression, obtained as copies/µl for ddPCR and CPM for RNAseq, was normalized on *TBP* as HKG. Relative expression was then normalized on D1R-Q20 samples (A) and on D2R-Q20 samples (B). Statistical significance was calculated via one-tailed t-test (α=0.05). For ddPCR, data are represented as mean ± SEM. **p<0,01, ***p<0,001. N=4 (Q20) and 5 (Q175) biological replicates. **C.** The scatter plot represents ddPCR and RNAseq expression data comparison via a correlation analysis. Expression obtained for every gene analyzed (*Actb, Drd1, Pdyn*, *Penk, Adora2a, Crym,* *Sfxn1, Atp5j2, Ndufa4, Ndufv3*, *Uqcc2, Rpl38*) in different conditions (Q20-D1R, Q20-D2R, Q175-D1R, Q175-D2R) was normalized on *TBP* as a housekeeping gene. Data was then represented as Log_2_(normalized expression). A high level of correlation between RNAseq and ddPCR results was obtained (Pearson R = 0,87, two-tailed t-test p-value = 4,21E-13, degrees of freedom: 38).

**Supplementary figure S7: PCA on single cells highlights distinct clustering of D1R and D2R-neurons. A.** The scatterplot represents an unbiased principal component analysis (PCA), which was initially performed on single cells gene expression. D1R- (red) and D2R- (blue) neurons tend to clusterize based on PC1. **B.** PC1 and PC2 marker genes are shown. **C.** D1R and D2R sample density, based on PC1 and PC2 from the PCA shown in **D.** Scatter plot reports PCA, performed on single cells by overlapping PCAs performed on Q20 and Q175 cells separately. This PCA illustrates separation between D1R (circles) and D2R cells (triangles), with D1R-neurons showing greater dispersion between Q175 (blue) and Q20 (green) conditions. PC1 explains 7.2% of variance while PC2 explains 4.2% of variance. PCs markers and their relative weights are shown below the PCA. **E.** The barplot represents PCA markers *Crym* and *Sfxn1* expression, detected via ddPCR in Q20 and Q175 D1R- and D2R-neurons. Gene expression was comparable (no significant differences) to RNAseq results. N=3 biological replicates. Gene expression, obtained as copies/µl for ddPCR and CPM for RNAseq, was normalized on *TBP* as a housekeeping gene. Relative expression was then normalized on D1R-Q20 samples. Statistical significance was calculated via one-tailed t-test (α=0.05). For ddPCR, data are represented as mean ± SEM.

**Supplementary figure S8: LINE-1 transposable elements (TEs) are down-regulated in Q175 neurons compared to Q20. A.** Volcano plot shows LINE-1 TEs, and L1MdF_II and L1MdF_III families being down-regulated in the disease condition compared to control. TEspeX was used on transcriptome data collected from small pools of MSNs to quantify and compare TEs (LINE-1, Young LINE-1 and others) expression in Q175 and Q20 neurons, without separating D1R- from D2R-expressing cells. **B.** The boxplot shows that expression of young LINE-1 elements specifically investigated in D1R and D2R neurons in healthy and disease condition, revealing a tendency toward lower levels in D1R for L1MdTf_I, II and III elements. Statistical analysis performed using DESeq2 (padj). *p<0,1, **p<0,05, ***p<0,001.

**Supplementary figure S9: MSNs do not present large copy number variations (CNVs). A.** CNVs analysis was performed in pools and single D1R- and D2R- neurons across the entire genome. Chromosomal regions with aberrations in at least two different samples are represented in the heatmap. A condition of diploidy (normal condition) is represented in white, while chromosomal duplications and ablations are represented respectively in red and blue. On the Y axis, samples are grouped per type: Q175-D1R, Q175-D2R, Q20-D1R, Q20-D2R. On the X axis, chromosomal regions with consecutive chromosomal aberrations are indicated and separated by chromosomal number. Overall, the samples present a condition of diploidy in almost the entire genome. Few chromosomal ablations and one chromosomal duplication were detected across the genome. However, the comparison with RNAseq data indicated that this signal is most likely due to background noise. **B.** In the heatmap, the number of chromosomal alterations (both ablations and duplications) were grouped per single chromosome (Y axis) and per sample type (X axis). The sum of chromosomal alterations is indicated on the bottom and on the left of the graph. While the majority of aberrations can be ascribable to chromosome 19, no significant differences were detected between different samples, indicating that the presence of CNVs is not related to cell type or the disease condition.

**Supplementary figure S10: Immunofluorescence image of a mouse striatal section.** Mosaic representative immunofluorescence image of a *Htt^Q20^* striatal section, positive for *Drd1*-TdTomato, *Drd2*-EGFP and Nuclei (ToPro3) stainings. The striatal section was virtually divided into four areas: dorsal-medial (I), dorsal-lateral (II), ventral-lateral (III) neostriatum, and ventral-medial neostriatum with a portion of the nucleus accumbens (IV). In the figures are indicated the corpus callosum (cc), the striatum (str), the cortex (ctx), the lateral ventricle (lv), nucleus accumbens (nac, delimited by dashed line) and the anterior part of the anterior commissure (aco). Cell counting was performed within the striatum region delimited by the cortex, the lateral ventricle, and the solid line. Scale bar: 200 µm.

**Supplementary figure S11: MSNs distribution and mutant huntingtin accumulation within striatal areas.** D1R- and D2R-MSNs proportion and mutant huntingtin accumulation within MSNs was evaluated into four striatal areas: dorsal-medial (I), dorsal-lateral (II), ventral-lateral (III) neostriatum, and ventral-medial neostriatum with a portion of the nucleus accumbens (IV), across different time points (4, 6 and >16 months of age), and represented in bar graphs. **A-C.** The MSNs proportion at 4, 6 and >16 months of age in *Htt^Q20^* mice didn’t reveal any significant differences but only a nearly significant tendency in having more D1R- than D2R-MSNs in region IV. **D-F.** D1R-MSNs are instead significantly greater in number compared to D2R in the entire *Htt^Q175^* striatum at 4 months. At 6 months, this difference is still visible only in the ventral striatum. At >16 months, no more significant differences are observed, but the same tendency is still detectable in region IV. A great decrease of both MSNs populations is present at this time point. MSNs proportion was calculated by normalizing the number of D1R- and D2R- cells over the total number of cells (nuclei). **G-I.** The amount of total (diffuse and aggregated) mutant huntingtin within D1R- and D2R- neurons does not differ across the *Htt^Q175^* striatum and between D1R- and D2R-MSNs, but at advanced disease stage the proportions of neurons presenting nuclear mutant huntingtin is decreased. **J-L.** In *Htt^Q175^* striatum, nuclear aggregated mutant huntingtin is borderline significantly more accumulated within D2R-MSNs compared to D1R at 4 months of age. This tendency is then lost at 6 and >16 months of age. The accumulation of nuclear mutant huntingtin within MSNs was calculated by dividing the number of D1R- or D2R-MSNs positive for EM48 by the total number of D1R- or D2R-MSNs. Statistical analysis was performed applying two-way ANOVA (ɑ=0.05) multiple comparison. ***p<0,001, *p<0,05. Number of biological replicates for every experiment are indicated in figure. Data are represented as mean ± SD.

**Supplementary figure S12: Neurodegeneration or cell death are not detectable in *Htt^Q175^* 6 months old mouse striatum. A.** The presence of cell death was evaluated in protein extracts from 6 months old *Htt^Q20^* and *Htt^Q175^* mouse striata using antibodies for cleaved (CL) Caspase 3, cleaved and full-length (FL) Parp, as shown in the representative western blot. Positive control for cell death is represented by NSC-34 cell line treated with Staurosporine. While Caspase 3 signal was completely undetectable from both the mouse genotypes, relative protein level expression of Parp obtained by quantifying WB image and normalizing on Gapdh as HKG didn’t reveal any significant difference between *Htt^Q20^* and *Htt^Q175^* mice. N = 4 biological replicates. **B.** The barplot represents autophagy marker *Lc3b* expression, evaluated on RNA extracted from 6 months old *Htt^Q20^* and *Htt^Q175^* mouse striata. Positive control for autophagy activation is represented by St*Hdh* Q^7/7^ cell line treated with rapamycin. Quantification of the gene expression (2^-ΔΔCt^ method, normalizing on *Pgk1* as HKG) showed no difference between the two genotypes. N = 4 biological replicates (mouse striata), N = 2 technical replicates for positive/negative controls. **C.** As shown on the left by representative western blot, autophagy levels were also evaluated in protein extracts from 6 months old *Htt^Q20^* and *Htt^Q175^* mouse striata using antibodies for CL and FL Gabarap. Hsp90 was used as HKG. On the right, the barplot shows autophagy levels in *Htt^Q20^* and *Htt^Q175^* mice, evaluated by calculating the ratio between CL and FL Gabarap. No significant differences were observed. N = 4 biological replicates. Statistical analysis was performed by applying two-tailed t-test (ɑ=0.05) and data are presented as mean ± SD.

**Bergonzoni et al., 2025 – Supplementary tables**

Table S1: DEGs

Table S2: Significant GO terms

Table S3: GO terms after semantic similarity

Table S4: DEGs D1R vs D2R Lee *et al.* 2020

Table S5: GO terms Lee *et al.* 2020

Table S6: Significant GO terms after semantic similarity Lee *et al.* 2020

Table S7: Shared GO terms Q20 vs WT (Lee *et al.* 2020)

Table S8: RBPs GO terms

Table S9: RBPs GO terms after semantic similarity

Table S10: TEs expression
